# Solubility Limit and Thermostructural Properties of Kagome Mg_3_V_2(1–*x*)_Nb_2*x*_O_8_: A Joint Diffraction and Spectroscopic Study

**DOI:** 10.1021/acs.inorgchem.6c00969

**Published:** 2026-08-18

**Authors:** Houri S. Rahimi Mosafer, Asiyeh Shokri, Iraida N. Demchenko, Anna Wolska, Marcin Klepka, Aleksandra Drzewiecka-Antonik, Alexey N. Shekhovtsov, Miron Kosmyna, Roman Minikayev, Wojciech Paszkowicz

**Affiliations:** † Institute of Physics, Polish Academy of Sciences, Al. Lotnikow 32/46, Warsaw 02-668, Poland; ‡ International Research Centre Magtop, Institute of Physics, Polish Academy of Sciences, Al.Aleja Lotniktow 32/46, Warsaw should be deletedPL-02-66802668, Poland; § Institute of Plasma Physics and Laser Microfusion, ul. Hery 23, Warsaw 01-497, Poland; ∥ The Centre for Advanced Materials and Technologies, CEZAMAT at the Warsaw University of Technology, 19 Poleczki Street, Warsaw 02-822, Poland; ⊥ Institute for Single Crystals, NAS of Ukraine, Nauky Ave. 60, Kharkov 61001, Ukraine

## Abstract

Modification of composition via solid solution can significantly improve the physical properties of materials. The crystallographic properties of the novel Mg_3_V_2(1–*x*)_Nb_2*x*_O_8_ solid solution were investigated by X-ray powder diffraction (XRPD) and spectroscopy. XRPD analysis confirms crystallization in the *Cmca* space group, corresponding to the kagome-staircase Ni_3_(VO_4_)_2_-type structure. Rietveld refinements reveal that Nb substitution induces a systematic increase in lattice parameters and unit cell volume. However, the lattice parameters remain constant for *x* > 0.04, indicating a solid solution limit of approximately *x* ∼ 0.04. Spectroscopic analysis further indicates tetrahedral coordination of Nb dopants, as evidenced by the characteristic energy positions and splitting of the Nb L_3_ edge spectra. High-temperature XRD studies (298–1273 K) revealed pronounced anisotropic thermal expansion. The volumetric thermal expansion coefficient increases from 19(2) MK^–1^ at 298 K to 40.6(5) MK^–1^ at 1273 K. Electronic structure calculations based on density functional theory yield a band gap of 3.57 eV, which is in good agreement with previously reported experimental data. The calculated Debye temperature (734 K) indicates high lattice rigidity. The observed trend in directional compressibility (*a* > *c* > *b*) correlates well with the experimentally measured anisotropic thermal expansion, confirming the *a*-axis as the most responsive under both thermal and mechanical conditions.

## Graphical abstract

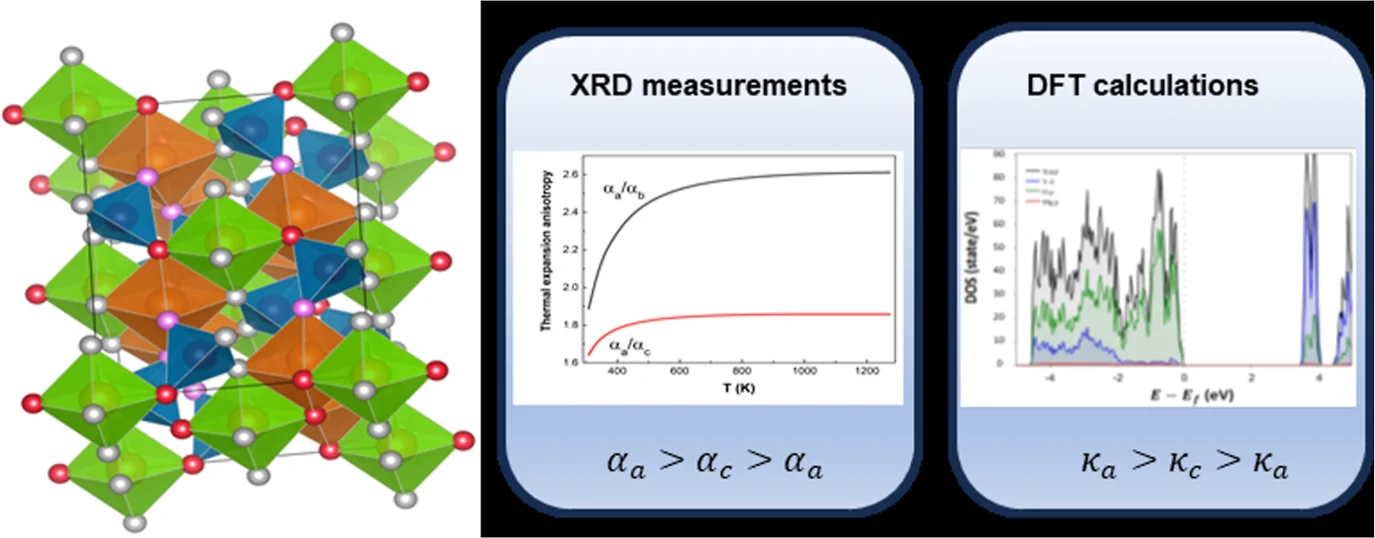

## Introduction

Magnesium orthovanadate, Mg_3_V_2_O_8_, has attracted significant attention, owing to its diverse functional properties. Mg_3_V_2_O_8_ crystallizes in an orthorhombic structure (space group *Cmca*, Z = 4) of the Ni_3_(VO_4_)_2_-type structure. Its structural properties were first studied in 1971.^1^ In this class of divalent-metal orthovanadates, the structure is formed by edge-sharing MO_6_ (M = Mg, Mn, Co, Ni, and Zn) octahedra arranged in two-dimensional kagome-type staircases. These layers are separated by oxyanion VO_4_ tetrahedra that share corner oxygen atoms with the MO_6_ octahedra (see Figure 1).

**Figure 1:**
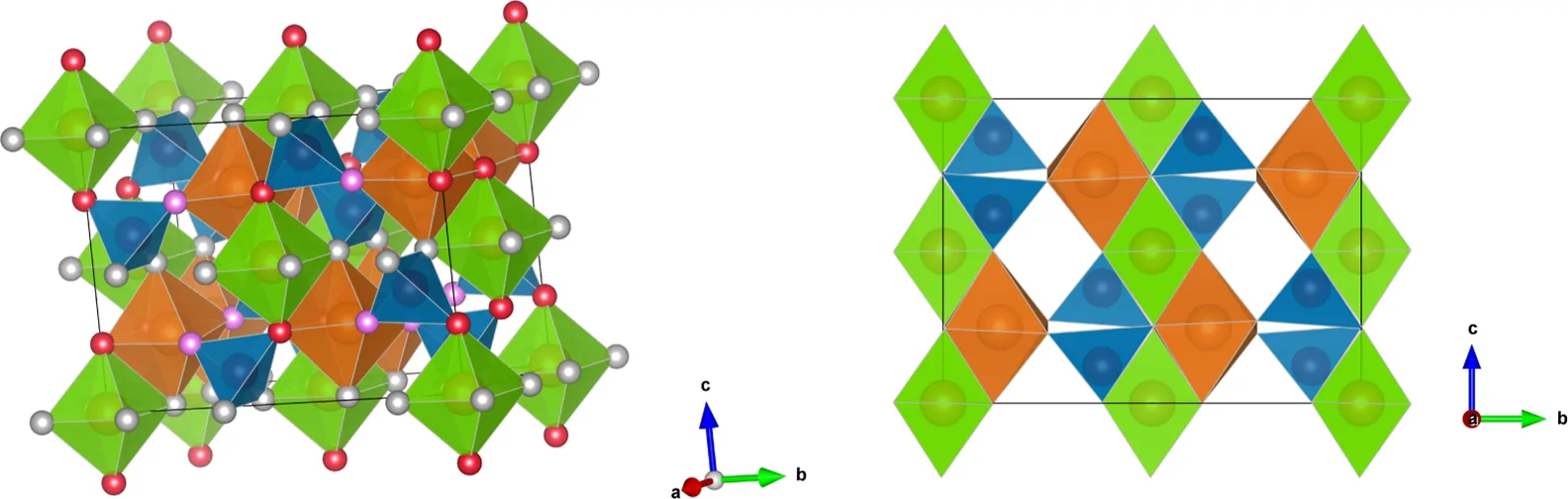
Crystal structure of Mg_3_V_2_O_8_, showing Mg(1)­O_6_ octahedra (green), Mg(2)­O_6_ octahedra (orange), and VO_4_ tetrahedra (blue). Oxygen atoms are labeled as O(1) (pink), O(2) (red), and O(3) (gray). The right figure shows a projection view of Mg_3_V_2_O_8_, highlighting the staircase arrangement.

The applications of magnesium vanadates are strongly influenced by their thermodynamic behaviors. In an earlier study, the free energy of formation of Mg_3_V_2_O_8_ was estimated over a wide temperature range (298–1200 K), demonstrating its stability under high-temperature conditions encountered in industrial applications, such as the mitigation of vanadium-induced hot corrosion in gas turbines.^2^

Materials with the Ni_3_(VO_4_)_2_-type structure, such as Mg_3_V_2_O_8_, have attracted considerable attention because of their catalytic, optical, and electrochemical properties. Mg_3_V_2_O_8_ has been investigated for catalytic and photocatalytic applications, including propane oxidative dehydrogenation and visible light-driven water splitting. In addition, studies on M_3_V_2_O_8_ (M = Ni, Zn) have shown that cation substitution strongly influences the electronic structure and catalytic performance.^3,4^ Its luminescence originates from electronic transitions in VO_4_ tetrahedra, and the emission is highly sensitive to local structural distortions.^5^ Changes in the M^2+^ cation modify tetrahedral geometry and emission behavior, making Mg_3_V_2_O_8_ a promising phosphor material.^6^ Nb^5+^ doping is expected to increase V–O bond lengths and induce a red shift, with potentially improved luminescence efficiency. In addition, Mg_3_V_2_O_8_ nanoparticles show enhanced charge transfer and energy storage performance when used in modified electrodes.^7^ Mg^2+^ transport in Mg_3_V_2_O_8_ has been studied by ^51^V NMR, revealing an activation energy of about 1.03 eV. The study also identified distinct cation hopping mechanisms determined by octahedral site connectivity.^8^

Recent advances in synthesis, including hydrothermal routes and controlled solid–state reactions, have further enabled the preparation of Mg_3_V_2_O_8_ with tailored morphologies and particle sizes.^9^ These morphological variations can influence both the optical band gap and the photocatalytic and photostrictive responses of the material, as reported for nanostructured Mg_3_V_2_O_8_.^9^

Divalent metal ion substitution at the M-site in M_3_V_2_O_8_ (M = Mg, Co, Ni, Zn, and Mn) significantly influences the structural and magnetic properties of the material. Studies on (Co_1–*x*_Mg_*x*_)_3_V_2_O_8_ show that increasing Mg content gradually alters the crystal growth direction and affects the magnetic behavior.^10^ Partial replacement of Ni^2+^ by Co^2+^ or Mg^2+^ in Ni_3_V_2_O_8_ modifies the magnetic ordering temperatures and introduces distinct spin structures. It can also change the magnitude of octahedral distortions in the lattice.^11,12^ Similarly, in Zn_3_V_2_O_8_, the substitution of divalent transition metals such as Co^2+^, Ni^2+^, or Cu^2+^ modifies the electronic and optical properties. Optical absorption studies indicate that these substitutions induce metal–metal charge transfer (MMCT) transitions. As a result, the band gap and color of the compounds are modified.^13^ For instance, Co^2+^ substitution shifts the absorption bands to lower energies due to its electronic interactions with V^5+^, while Ni^2+^ substitution introduces distinct electronic states that modify the optical transitions. These effects highlight the ability to tune functional properties in M_3_V_2_O_8_ compounds through controlled divalent cation substitution.

In addition to the M^2+^ site, the vanadium site can also be substituted by other ions, such as Nb^5+^. In recent years, substituting niobium (Nb^5+^) for vanadium (V^5+^) in orthovanadate compounds has emerged as a promising strategy for tailoring structural, electronic, and optical properties. For instance, in whitlockite materials such as Ca_9_Y­(VO_4_)_7_ and Ca_9.75_Eu_0.5_(VO_4_)_7_, Nb substitution improves the photoluminescence properties and makes these compounds promising candidates for nonlinear optical applications.^14,15^ Nb doping in Li_3_VO_4_ has been demonstrated to enlarge lattice parameters and enhance Li-ion diffusion, thereby boosting electrochemical performance as an anode material for lithium-ion batteries.^16^ In LaV_1–*x*_Nb_*x*_O_4_, Nb substitution drives a transformation from a monoclinic-monazite to a scheelite-tetragonal phase. This transformation is accompanied by significant changes in vibrational and electronic characteristics, enhancing the luminescence and dielectric properties.^17,18^ Similar effects are observed in (V_1–*x*_Nb_*x*_)­OPO_4_ solid solutions, where Nb substitution leads to less distorted octahedral environments and improved catalytic behavior.^19^ In kagome superconductors like Cs­(V_1–*x*_NB_*x*_)_3_Sb_5_, Nb substitution shifts key electronic bands to enhance superconducting transition temperatures while suppressing competing charge density waves.^20^

While Mg_3_V_2_O_8_ has been widely studied for its multifunctional properties, including luminescence, catalysis, and ionic transport, its thermal expansion behavior and thermostructural stability at high temperatures remain unexplored. Furthermore, despite the extensive work on divalent cation substitution at the M^2+^ site, substitution at the V^5+^ site, particularly with Nb^5+^, has received considerably less attention. Nb^5+^, with its larger ionic radius and more spatially extended 4d orbitals compared to those of V^5+^, is known to induce structural relaxation, enhance ionic conductivity, and tune the band structure in various oxide systems. Substitution of Nb into Mg_3_V_2_O_8_ presents a promising approach for controlling the local structural parameters, electronic properties, and luminescence characteristics. This study aims to investigate the effects of Nb substitution at the vanadium site on the thermostuctural and elastic properties of Mg_3_V_2_O_8_.

## Methods

### Experimental Details

Solid solutions Mg_3_V_2(1–*x*)_Nb_2*x*_O_8_ with varying Nb content were produced by means of solid state synthesis. Predried initial reagents Mg­(OH)_2_ (Alfa Chemistry, 99.99%), V_2_O_5_ (Merck, 99.99%), and Nb_2_O_5_ (Merck, 99.99%) were used for synthesis according to the following reaction.

$$\begin{array}{l} 3Mg{\left(OH\right)}_{2}+\left(1-x\right)V_{2}O_{5}+{xNb}_{2}O_{5}\to {Mg}_{3}V_{2\left(1-x\right)}{Nb}_{2x}O_{8}+3H_{2}O↑\\ x=0.01,0.02,0.03,0.04,0.1 \end{array}$$

The synthesis procedure was carried out in air by using a resistive furnace and consisted of two steps. Stoichiometric mixtures of reagents were placed in Pt crucibles, heated to 873 K at a rate of 50 K/h, and kept for 17 h. After that, mixtures were reground, heated again to 1423 K at a rate of 150 K/h, and kept for 36 h. The cooling rate was consistent for both stages −150 K/h.

Room temperature (RT) X-ray powder diffraction (XRPD) measurements were performed using a Philips X’Pert Pro Alpha1 diffractometer with Cu Kα_1_ radiation, in Bragg–Brentano geometry and operated in continuous scanning mode. The instrument was equipped with a linear silicon strip detector and an incident beam Ge(111) Johansson monochromator. The detector configuration principles were based on ref 21, with instrument setting details as reported in ref 22. XRPD data were collected at RT over the 2θ range of 13.5–159.2°, with a step of 0.0167°. Crystal structures were refined using the Rietveld method^23^ using the Fullprof (version April 2019) software.^24^ High-temperature diffraction measurements were conducted using the same diffractometer equipped with an HTK 1200N (Anton Paar, Graz, Austria) temperature stage, in the Bragg–Brentano geometry with CuKα radiation. The high-temperature XRPD data were collected over the 2θ range of 14.5–110°. The set temperature range from RT (298 K) to 1273 K was selected, with different temperature steps (usually 50 or 100 K).

The X-ray absorption near-edge structure (XANES) measurements were performed at the ASTRA beamline at the SOLARIS synchrotron (Krakow, Poland). The Nb L_3,2_-edges for the powdered samples were measured in the fluorescence mode at RT. The XANES data were handled and analyzed using the Demeter package.^25^ The peak-fitting procedure was used during analysis. For all considered spectra, *E*_0_ was set at 2371 eV. The line for background removal was defined within the range of 2323 eV–2350 eV. The normalization range was 2415 eV–2460 eV, with limitations resulting from the close presence of the L_2_ edge. The fitting window was 2365 eV–2388 eV.

### Computational Details

To gain insight into the structural and electronic properties of Mg_3_V_2_O_8_, density functional theory (DFT) calculations were performed. The calculations were designed to validate the structural model, including lattice parameters, symmetry, and band gap, and to examine the structural response of an idealized single-phase solid solution upon Nb substitution at the V site.

All calculations were carried out using the projector-augmented wave (PAW) method as implemented in the Vienna Ab initio Simulation Package (VASP).^26–29^ The exchange–correlation functional dependence was systematically evaluated using three approaches: the generalized gradient approximation (GGA), the SCAN *meta*-GGA functional,^30^ and a modified B3LYP hybrid functional.^31^

The SCAN functional was used for structural optimization and electronic property analysis because of its established reliability for systems containing localized electrons, particularly in predicting lattice parameters and band gaps with improved treatment of intermediate-range exchange–correlation effects. The GGA functional, known to overestimate lattice constants and underestimate band gaps, was used only as a baseline reference for benchmarking structural and electronic trends.

For mechanical properties, a hybrid B3LYP functional with 40% exact-exchange mixing was employed alongside SCAN in order to compare their descriptions of elastic response and anisotropic compressibility. In the B3LYP formulation, the exchange–correlation energy is constructed as a linear combination of Hartree–Fock exchange and density functional exchange–correlation terms, with the exact-exchange fraction set to 0.40. This modified hybrid scheme is used specifically to evaluate energy-strain relationships and elastic response within the harmonic approximation. It should be emphasized that this functional choice is intended for mechanical property trends rather than full electronic structure accuracy. As previous studies have demonstrated, B3LYP with 40% exact-exchange mixing better captures “energy-strain” responses and elastic properties under ambient pressure.^32^ The comparison between the calculated elastic anisotropy and experimentally observed thermal expansion behavior should be interpreted in a qualitative sense due to the neglect of finite-temperature anharmonic effects.

Elastic constants were calculated using the energy-strain method. Small symmetry-preserving strains in the range of -0.015 to +0.015 (±1.5%) were applied to the optimized structure, and the resulting energy-strain response was used to extract the elastic tensor via linear fitting. The strain amplitudes were chosen to remain within the harmonic regime. Elastic tensors were postprocessed using VASPKIT.^33^

A plane-wave kinetic energy cutoff of 520 eV was used, and Brillouin zone integrations were performed using an 8 × 4 × 6 Monkhorst–Pack *k*-point mesh. For both structural relaxation and elastic constant calculations, electronic convergence was set to 10^–6^ eV, and ionic relaxations were stopped when forces were below 0.01 eV/Å.

## Results and Discussion

### Structural Study at Room Temperature as a Function of Nb Content

The phase analysis based on XRPD patterns confirms the formation of Mg_3_V_2(1–*x*)_Nb_2*x*_O_8_ with the *Cmca* space group. A trace of β-Mg_2_V_2_O_7_ impurity phase, with the *P*1̅ space group, is also detected. Rietveld refinements were performed for all samples using the structure of Mg_3_V_2_O_8_ ^1^ as the initial model. Peak shapes were modeled using the pseudo-Voigt type and considering two asymmetry parameters. Furthermore, isotropic atomic displacement factors were set to be identical for all oxygen atoms. A total of 33 parameters were refined: scale factor (1), lattice parameters (3), atomic coordinates (10), thermal displacement factors (4), systematic error (1), peak shape (5), peak asymmetry (2), and background (7). Due to the high data quality, seven parameters (scale factor and lattice parameters) of the second phase were refined. The lattice parameters of the minor phase, Mg_2_V_2_O_7_, closely matched those reported in ref 34. Additionally, structural data for the MgNb_2_O_6_ (*Pbcn* space group), which appears in the sample with *x* = 0.1, were taken from ref 35.

The obtained unit cell volume of Mg_3_V_2_O_8_ is close to that reported in ref 1, with a discrepancy of approximately 0.05%. The final results of refinements are summarized in Tables 1 and 2. The quality of the refinements is illustrated for Mg_3_V_1.96_Nb_0.04_O_8_ at 298(2) K (Figure 2) as a representative example. Additional refinement plots for the other compositions are presented in the Supporting Information, Figures S1–S5.

**Table 1:** Structural Details of Mg_3_V_2(1–*x*)_Nb_2*x*_O_8_ (Lattice Parameters, Volume, and Density) at 298 K

*x*	*a* (Å)	*b* (Å)	*c* (Å)	*V* (Å^3^)	ρ (g cm^–3^)
0	6.06195(2)	11.44016(5)	8.31465(3)	576.618(4)	3.488
0.01	6.06310(3)	11.44450(6)	8.31692(4)	577.104(5)	3.495
0.02	6.06406(2)	11.44991(4)	8.31826(3)	577.562(4)	3.502
0.03	6.06406(4)	11.45098(7)	8.32047(5)	577.769(6)	3.510
0.04	6.06481(3)	11.45783(6)	8.32403(4)	578.433(5)	3.516
0.1^a^	6.06505(8)	11.45669(15)	8.32336(10)	578.35(1)	3.574

a *x* = 0.1 refers to the nominal composition, while the actual Nb content corresponds to the solubility limit.

**Table 2:** Refined Atomic Positions (*x*, *y*, *z*) of Mg_3_V_2(1–*x*)_Nb_2*x*_O_8_ at 298 K

			*x*				
site	Wyckoff	coordinate	0	0.01	0.02	0.03	0.04
Mg1	4a	*x*	0	0	0	0	0
		*y*	0	0	0	0	0
		*z*	0	0	0	0	0
Mg2	8e	*x*	0.25	0.25	0.25	0.25	0.25
		*y*	0.1349(2)	0.1348(2)	0.1357(1)	0.1335(2)	0.1350(1)
		*z*	0.25	0.25	0.25	0.25	0.25
V	8f	*x*	0	0	0	0	0
		*y*	0.37987(6)	0.38012(7)	0.38049(5)	0.38012(7)	0.38023(6)
		*z*	0.12127(9)	0.1206(1)	0.12116(9)	0.1208(1)	0.12100(9)
O1	8f	*x*	0	0	0	0	0
		*y*	0.2509(2)	0.2512(3)	0.2511(2)	0.2495(3)	0.2505(3)
		*z*	0.2289 (2)	0.2303(3)	0.2302(2)	0.2324(3)	0.2307(2)
O2	8f	*x*	0	0	0	0	0
		*y*	0.0042(2)	0.0031(3)	0.0045(2)	0.0048(3)	0.0052(3)
		*z*	0.2446(2)	0.2425(3)	0.2433(2)	0.2426(3)	0.2421(2)
O3	16g	*x*	0.2744(2)	0.2746(3)	0.2734(2)	0.2741(3)	0.2734(2)
		*y*	0.1190(2)	0.1190(2)	0.1170(1)	0.1186(2)	0.1184(2)
		*z*	0.9970 (2)	0.9960(2)	0.9976(2)	0.9962(2)	0.9963(2)

**Figure 2:**
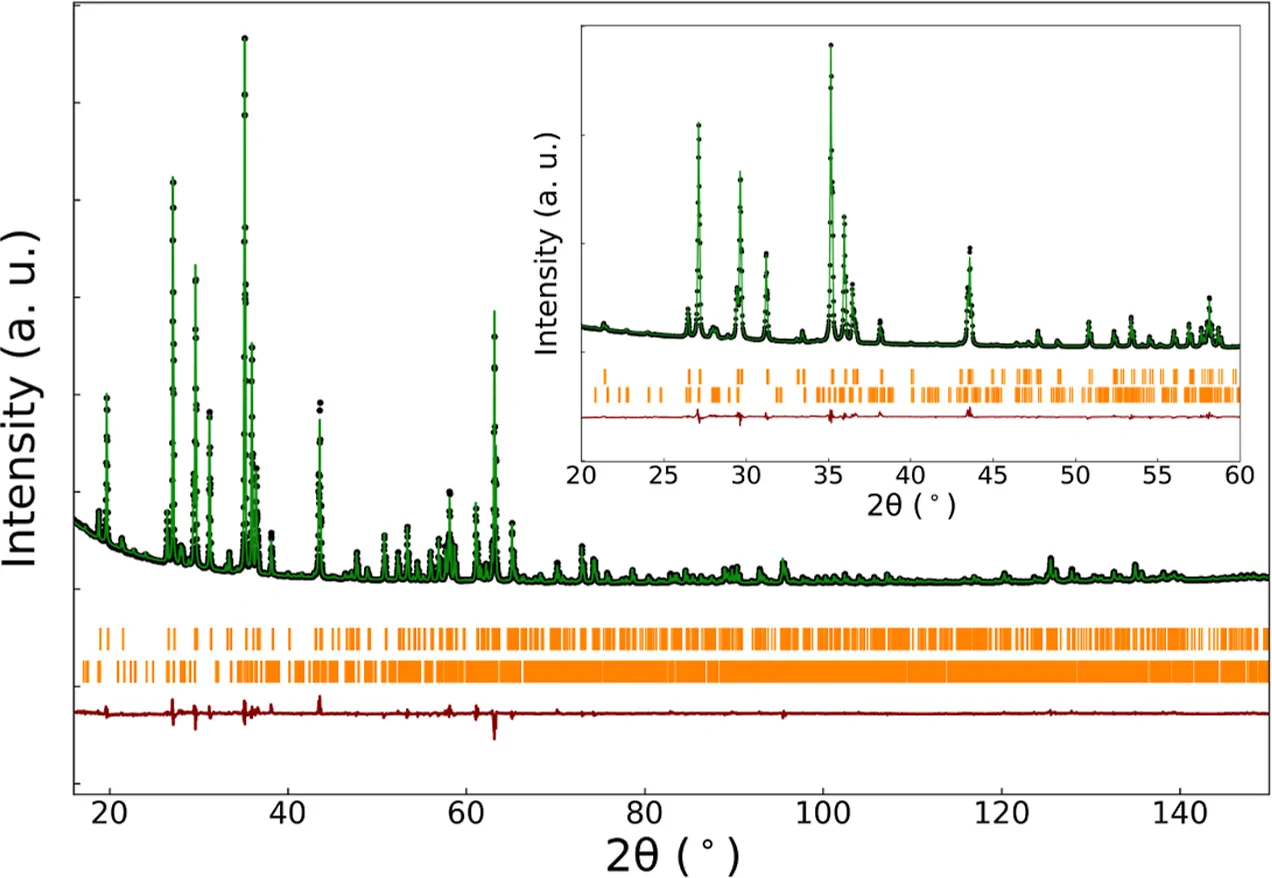
Illustration of Rietveld refinement of Mg_3_V_1.96_Nb_0.04_O_8_ at 298 K. Black circles correspond to the experimental data, the green line to the calculated profile, orange ticks to Bragg reflections (upper: Mg_3_V_1.96_Nb_0.04_O_8_-main phase and lower: Mg_2_V_2_O_7_-minor phase), and the red line to the residual. *R*_p_ = 2.98%, *R*_wp_ = 3.96%.

The refined lattice parameters increase with the substitution of Nb ions. The increase in lattice parameters *a*, *b,* and *c* and consequently unit cell volume, with increasing Nb content, provides evidence for Nb substitution into the Mg_3_V_2_O_8_ (see Figure 3). The increase in unit cell size is attributed to the difference in ionic radii between V^5+^ (0.355 Å) and Nb^5+^ (0.48 Å).^36^

**Figure 3:**
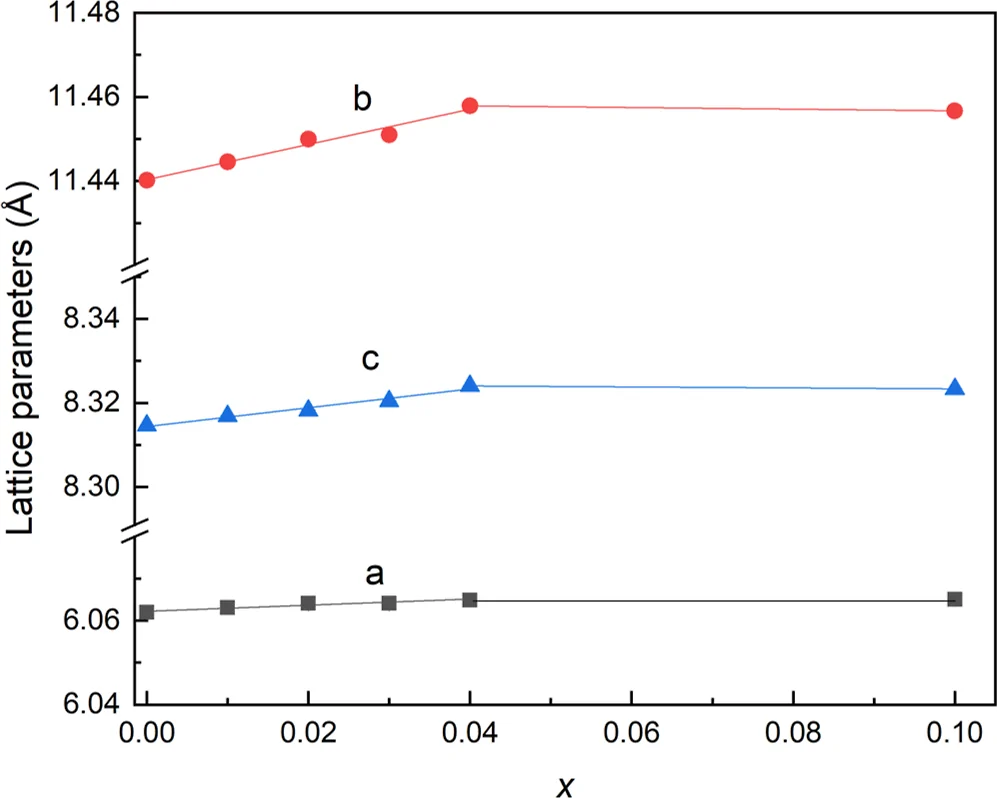
Evolution of lattice parameters (*a*, *b*, *c*) with Nb substitution in Mg_3_V_2_O_8_.

In Mg_3_V_2(1–*x*)_Nb_2*x*_O_8_, the lattice parameters and unit-cell volume increase systematically up to *x* = 0.04 and remain nearly unchanged at higher nominal Nb contents. This trend indicates incorporation of Nb into the crystal structure up to at least *x* = 0.04. Samples with *x* = 0–0.04 contain the main phase together with Mg_2_V_2_O_7_ as a secondary phase, whereas the sample with *x* = 0.10 shows an additional Nb-containing phase, identified as MgNb_2_O_6_. Quantitative phase fractions for all compositions are summarized in Table S1. On this basis, the Nb solubility limit in Mg_3_V_2_O_8_ is at least about *x* = 0.04. As shown in Table 1, the unit-cell volume of the main phase increases systematically up to the composition containing 4 at % Nb, with an average change of ⟨Δ*V*⟩ = 0.45 Å^3^ per percentage point of Nb. Given the high accuracy of the lattice-parameter determination from X-ray diffraction data, the difference between the unit-cell volumes of the host phase at 4 at % and 10 at % Nb is only 0.08 Å^3^, which is more than five times smaller than ⟨Δ*V*⟩ per 1% Nb. Therefore, the Nb solubility limit is estimated to be approximately 4.0(3)%, although a more precise value cannot be established from the present data. This limitation arises from the compositional gap between *x* = 0.04 and 0.10, as well as from the limited reliability of V/Nb occupancy refinement by X-ray diffraction because of their similar scattering factors. A similar solubility limit is observed in Ca_9_Y­(VO_4_)_7_, where partial substitution of V^5+^ by Nb^5+^ is possible only up to ∼4 at %; beyond this, secondary Y_3_NbO_7_ phases form.^37^

In complex oxides, variations in bond lengths provide key insights into the structural effects of cation substitution. In Mg_3_V_2(1–*x*)_Nb_2*x*_O_8_, the Mg–O bond lengths remain nearly unchanged with increasing Nb content, indicating the structural stability of the MgO_6_ octahedra. Conversely, a slight elongation of the V–O bonds is observed upon substitution of V by Nb. (Figure 4). This trend is consistent with the difference in ionic radii of Nb^5+^ and V^5+^ in tetrahedral coordination, where the larger Nb^5+^ ion induces an expansion of the V–O bonds.

**Figure 4:**
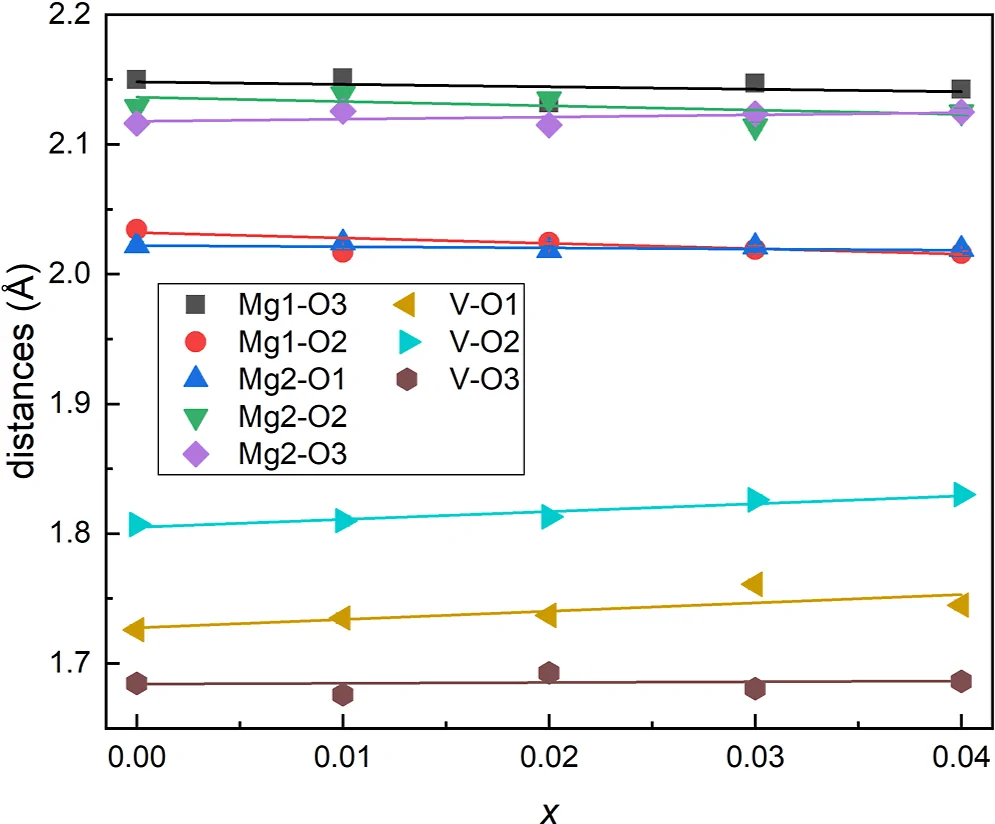
Variation of interatomic distances in Mg_3_V_2(1–*x*)_Nb_2*x*_O_8_ as a function of Nb substitution.

The bond distances for Mg_3_V_2_O_8_, as summarized in Table 3 reveal that its Mg–O bond distances (approximately 2.09 to 2.13 Å) are in close agreement with values reported for chemically related compounds such as Ca_5_Mg_4_V_6_O_24_ ^38^ and LiMgVO_4_.^39^ Similarly, the V–O distances in Mg_3_V_2_O_8_ exhibit a uniform tetrahedral coordination, aligning with the chemical trends observed in comparable systems. This close similarity confirms that the local geometry in Mg_3_V_2_O_8_ is consistent with related compounds such as Ca_5_Mg_4_V_6_O_24_,^38^ AgMgVO_4_,^40^ and BaMg_2_V_2_O_8_.^40^

**Table 3:** Comparison of Average Bond Length and Polyhedra Volume for Mg_3_V_2_O_8_ with Literature Values and Chemically Related Materials

	Mg		V		
samples	average distances	polyhedral volume	average distances	polyhedral volume	ref
Mg_3_V_2_O_8_	2.1113	12.2886	1.7256	2.6311	present study
	2.0892	12.0560			
Mg_3_V_2_O_8_	2.0983	12.0664	1.7256	2.6470	^1^
	2.0916	12.0884			
Mg_3_V_2_O_8_	2.1294	12.6238	1.7140	2.5776	^6^
	2.0888	12.0664			
Ca_5_Mg_4_V_6_O_24_	2.0837	12.0592	1.7347	2.6435	^38^
AgMgVO_4_	2.1641	13.0932	1.7212	2.5983	^40^
LiMgVO_4_	2.1703	13.5535	1.6589	2.3280	^39^
BaMg_2_V_2_O_8_	2.1001	12.2841	1.7189	2.8967	^41^
CuMgVO_4_	2.1075	12.3764	1.7354	2.6769	^40^

**Table 4:** Refined Atomic Positions (*x*, *y*, *z*) of Mg_3_V_2_O_8_ at Temperatures of 473, 673, 873, 1073, and 1273 K

		*T* (K)				
site	coordinate	473	673	873	1073	1273
Mg1	*x*	0	0	0	0	0
	*y*	0	0	0	0	0
	*z*	0	0	0	0	0
Mg2	*x*	0.25	0.25	0.25	0.25	0.25
	*y*	0.1344(3)	0.1337(4)	0.1346(4)	0.1361(4)	0.1360(4)
	*z*	0.25	0.25	0.25	0.25	0.25
V	*x*	0	0	0	0	0
	*y*	0.3799(1)	0.3799(2)	0.3800(2)	0.3807(1)	0.3808(2)
	*z*	0.1209(2)	0.1206(2)	0.1214(2)	0.1216(2)	0.1216(2)
O1	*x*	0	0	0	0	0
	*y*	0.2514(6)	0.2507(7)	0.2511(7)	0.2512(6)	0.2507(8)
	*z*	0.2307(6)	0.2338(6)	0.2323(6)	0.2297(6)	0.2277(6)
O2	*x*	0	0	0	0	0
	*y*	0.0050(6)	0.0053(7)	0.0030(7)	0.0041(6)	0.0013(8)
	*z*	0.2424(6)	0.2422(6)	0.2433(6)	0.2446(6)	0.2406(7)
O3	*x*	0.2763(5)	0.2757(6)	0.2789(6)	0.2787(5)	0.2812(6)
	*y*	0.1175(3)	0.1173(4)	0.1171(4)	0.1170(3)	0.1168(4)
	*z*	0.9952(4)	0.9956(4)	0.9940(4)	0.9937(4)	0.9920(5)

The substitution of Nb increases the volume of the VO_4_ tetrahedra. Simultaneously, there is a reduction in the volume of the Mg(1)­O_6_ octahedra, whereas the volume of Mg(2)­O_6_ octahedra remains unchanged (see Figure S6). This effect can be attributed to the shared O(3) oxygen atom between the Mg(1)­O_6_ octahedra and the VO_4_ tetrahedra. An increase in tetrahedral volume is accompanied by a reduction in the volume of the Mg(1)­O_6_ octahedra. Variations in polyhedral volume are linked to modifications in bond lengths and angles, and these distortions are represented by the distortion indices,^42^ which measure deviations of bond lengths and angles from their mean values. By substitution of Nb into Mg_3_V_2_O_8_, the distortion index of the tetrahedron increases from 0.024 to 0.029, whereas the octahedral distortion indices remain nearly constant at about 0.022 for Mg(2)–O and 0.025 for Mg(1)–O. The same behavior of increasing distortion in the tetrahedron also appears with Nb substitution into Ca_9_Y­(VO_4_)_7_.^14^ A significant increase in distortion index is associated with enhaced optical nonlinearity.^43^

The Nb L_3_-edge X-ray absorption spectra (XAS) provide valuable insights into the local coordination environment of the Nb ions in the investigated samples. The white line splits into a double structure, peaks A and B (see Figure 5), with another peak at higher energy, peak C. The A and B peaks correspond to the ligand field splitting of d orbitals, which originates from the local coordination around Nb ions, while peak C represents transitions from 2p_3/2_ to the Nb 5s state.^44^ The relative intensity and energy separation between peaks A and B differ significantly with the coordination geometry, octahedral versus tetrahedral.^45^ Specifically, for octahedral coordination, the energy gap is greater, typically close to or exceeding 3 eV, while for tetrahedral coordination, it is closer to 2 eV. Additionally, peak A tends to exhibit a lower intensity in tetrahedral environments. Similar trends were reported for Mo-based compounds.^46,47^

**Figure 5:**
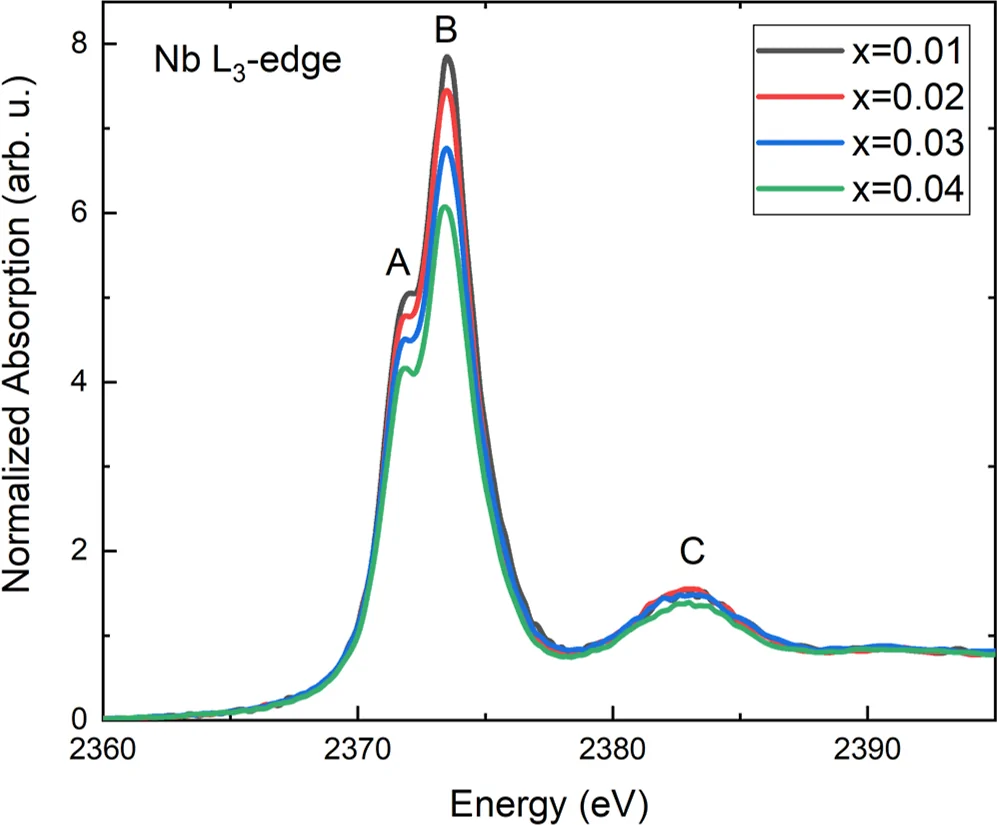
XANES spectra at the Nb L_3_ edge of Mg_3_V_2(1–*x*)_Nb_2*x*_O_8_ crystals.

Peak fitting of the Mg_3_V_2(1–*x*)_Nb_2*x*_O_8_ spectra was performed under identical conditions for all samples. Peaks A and B were fitted with Lorentzian functions, whereas peak C was fitted with a Gaussian function. In all samples, the peak positions were approximately 2371.6 eV for peak A, 2373.6 eV for peak B, and 2383.5 eV for peak C. The energy gap between peak A and peak B is equal to 2 eV, and the relative intensity of peak B is higher than for peak A. Both observations confirm a tetrahedral coordination environment for the Nb atoms.

### Crystal Structure Evolution in the Temperature Range 298–1273 K

High-temperature measurements were performed for samples of *x* = 0, 0.02, and 0.04. For all samples, the diffraction patterns show no phase transition or symmetry change in the temperature range from 298 to 1273 K. With increasing temperature, the diffraction peaks shift slightly toward lower angles due to lattice thermal expansion. The lattice parameters of Mg_3_V_2(1–*x*)_Nb_2*x*_O_8_ increase smoothly with temperature (see Figure 6). The *a* lattice parameter expands by 2% [from 6.06235(5) Å to 6.17979(7) Å], *b* by 0.7% [from 11.4397(2) Å to 11.5250(1) Å], and *c* by 1% [from 8.3150(1) Å to 8.4012(1) Å] over the range of 298–1273 K. Similar temperature behavior of lattice parameters has been reported for Zn_3_V_2_O_8_.^48^The refined atomic coordinates at selected temperatures are presented in [Table tbl4].

**Figure 6:**
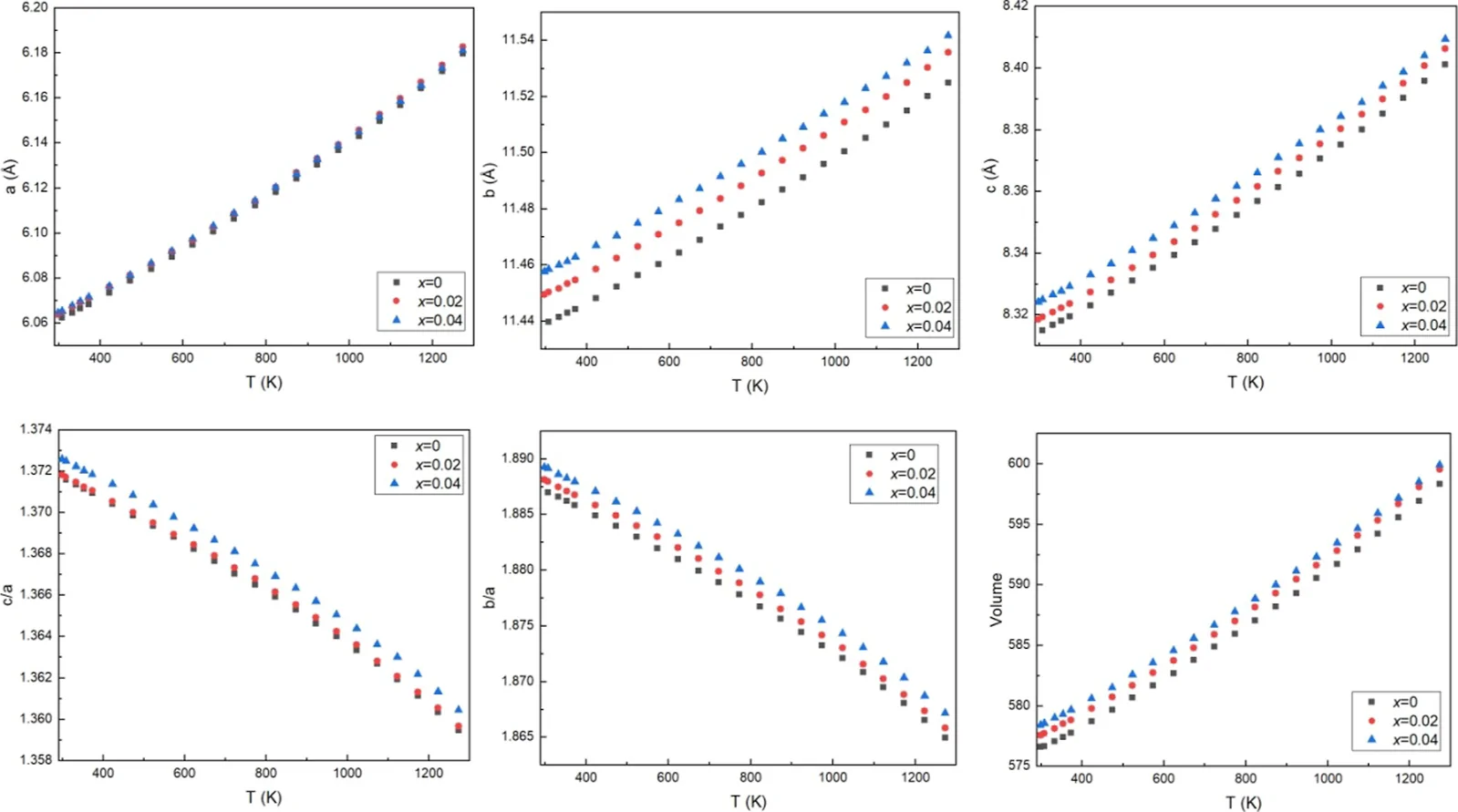
Variation of lattice parameters (*a*, *b*, *c*), axial ratio (*c*/*a*, *b*/*a*), and volume (*V*) with temperature for Mg_3_V_2(1–*x*)_Nb_2*x*_O_8_.

In order to calculate the thermal expansion coefficient, an empirical Laurent function was applied for fitting the unit cell size data in the range of 298–1273 K. The function used was similar to those previously described in the literature.^49,50^ The volumetric thermal expansion coefficient (α_V_) of the Mg_3_V_2_O_8_ is 19(2) MK^–1^ at 298 K and, with rising temperature, increases up to 40.6(5) MK^–1^ at 1273 K (see Figure 7). The volumetric thermal expansion at RT for Co_3_V_2_O_8_ and Ni_3_V_2_O_8_ is reported to be 22.5 and 24 MK^–1^, respectively.^51^ The mean value of volumetric thermal expansion of Zn_3_V_2_O_8_ in the temperature range 298–873 K was 54.3 MK^–1^.^48^ These variations may be associated with differences in bond stiffness as well as electronic and magnetic interactions.

**Figure 7:**
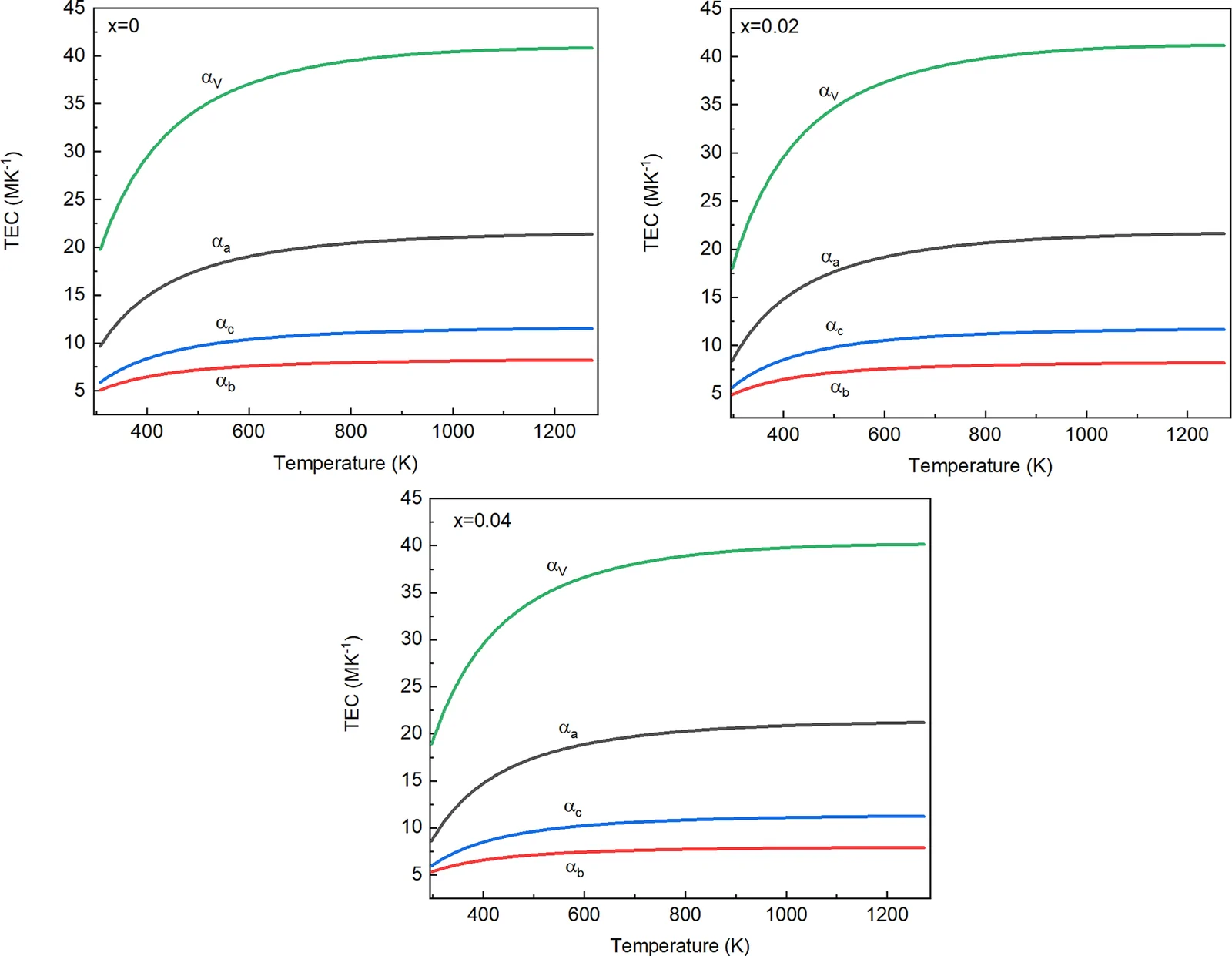
Temperature dependence of the volumetric thermal expansion coefficient of Mg_3_V_2(1–*x*)_Nb_2*x*_O_8_.

A similar trend is observed for compounds substituted with Nb, and within the method’s precision, the thermal expansion values show no significant difference. These phenomena can be attributed to the fact that the volume of the VO_4_ tetrahedra remains nearly constant with increasing temperature, indicating that thermal expansion is primarily driven by the Mg(1)­O_6_ and Mg(2)­O_6_ octahedra (see Figure 8). Since Nb substitutes at the V site, it has minimal influence on thermal expansion. Therefore, effective tuning of thermal expansion is expected to be achieved through targeted doping at the divalent (Mg) sites. The thermal expansion behavior of these materials is consistent with related vanadates, such as ScVO_4_ ^52^ and LuVO_4_,^53^ where the rigid nature of the VO_4_ oxyanion minimizes its contribution to overall thermal expansion. This temperature-invariant behavior of rigid tetrahedral units was also observed in PO_4_ ^52,53^ and AsO_4_ ^54^ tetrahedra. However, BeO_4_ tetrahedra^55^ exhibit notable volume expansion with increasing temperature, resulting in a measurable contribution to the unit cell’s thermal expansion.

**Figure 8:**
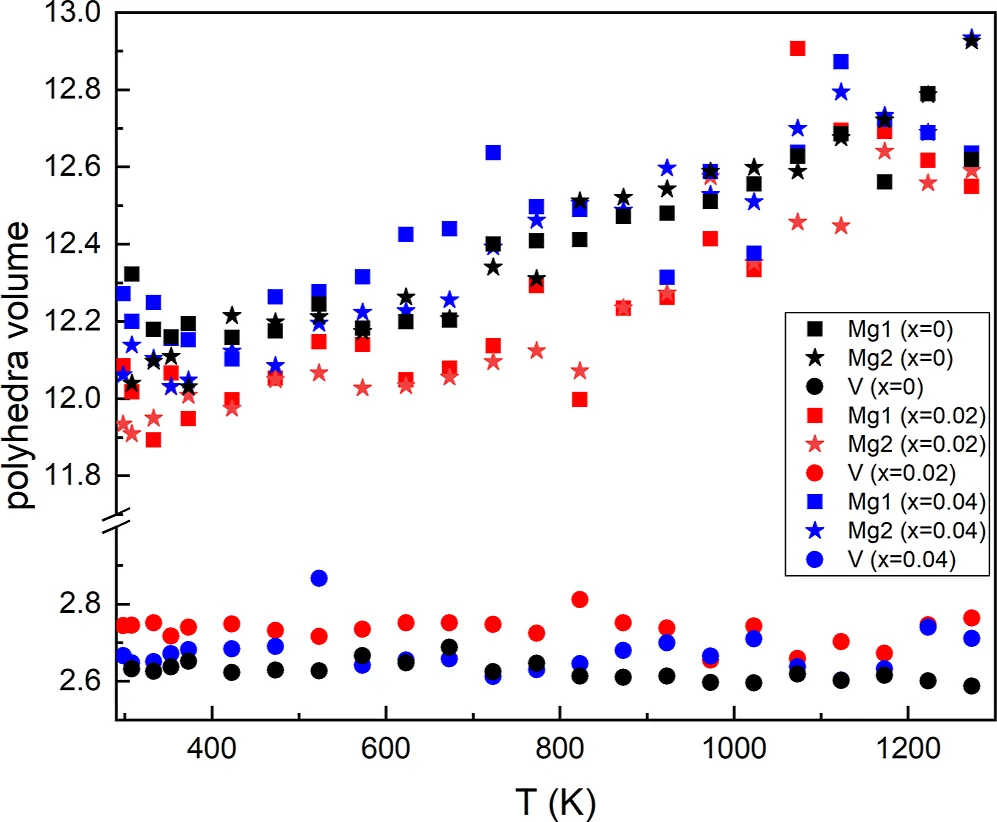
Variation of polyhedra volume with temperature for Mg_3_V_2(1–*x*)_Nb_2*x*_O_8_.

The ratios of thermal expansion anisotropy along different crystallographic axes $\left(\frac{\alpha_{a}}{\alpha_{b}}\;\text{and}\;\frac{\alpha_{a}}{\alpha_{c}}\right)$ in Mg_3_V_2_O_8_ range from 1.88 to 2.61 and 1.64 to 1.85, respectively (see Figure S7). These values indicate a pronounced anisotropy, with α_*a*_ > α_*c*_ > α_*b*_. These values confirm a pronounced anisotropy in thermal expansion, with the expansion along the *a*-axis being the most significant, followed by the *c*- and *b*-axes. A similar pattern is observed with Nb substitution. Mg(2) polyhedra expand isotropically in all three directions, while Mg(1) shows a more pronounced expansion along the *a*-axis, which is consistent with thermal expansion anisotropy. This correlation is maintained in substituted samples up to approximately 1000 K. Beyond this temperature, the correlation effects disappear, indicating a possible change in the mechanism, which requires further investigation.

A similar anisotropic deformation behavior was revealed from the DFT calculations performed in this study, indicating that the compressibility along the *a*-axis is greater than along the *b*- and *c*-axes for Mg_3_V_2_O_8_. This result is consistent with previous first-principles studies on related M_3_V_2_O_8_ compounds (M = Zn, Ni, and Mg).^32,56^ However, a high-pressure experimental study reported a slightly different trend for Mg_3_V_2_O_8_, with κ_*c*_ > κ_*a*_ > κ_*b*_, suggesting some variation in compressibility anisotropy under extreme conditions.^57^

### Computational Results

The lattice parameters and band gap values of Mg_3_V_2_O_8_ were obtained with the GGA, SCAN, and B3LYP functionals (see Table 5). As expected, GGA overestimates the lattice constants and severely underestimates the band gap. SCAN provides good agreement with experimental data for both structural parameters and the electronic band gap, supporting the reliability of describing the electronic structure of the material. SCAN yields an indirect band gap of 3.57 eV, which is consistent with reported experimental and theoretical values.^9,58^ The good agreement between the SCAN-calculated lattice parameters and the band gap of Mg_3_V_2_O_8_ and the corresponding XRD results establishes a reliable computational reference for analyzing substitution-induced structural trends within the *Cmca* phase.

**Table 5:** Calculated Optimized Lattice Constants (Å), Volume, and Energy Gap (eV) of Mg_3_V_2_O_8_ Calculated using GGA and *Meta*-GGA (SCAN) Methods, Compared with Available Experimental Data

method	*a* (Å)	*b* (Å)	*c* (Å)	*V* (Å^3^)	*E*_g_ (eV)	ref
*Meta*-GGA (SCAN)	6.008	11.429	8.270	567.88	3.57	this work
GGA	6.103	11.549	8.383	590.93	3.27	this work
PBE	6.1033	11.5556	8.3793	590.96	3.3	^58^
B3LYP	5.9061	11.2516	8.1216	539.705		^32^
GGA	6.11	11.57	8.40	593.24	3.34	^59^
experiment	6.06195(2)	11.44016(5)	8.31465(3)	576.618(4)		this work
experiment	6.053(3)	11.442(6)	8.330(3)	576.92		^1^
experiment					3.5	^9^
experiment	6.060(2)	11.424(4)	8.304(3)	574.88(5)		^6^
experiment	6.05	11.44	8.33	576.92	3.15	^59^

As can be seen in the projected density of states shown in Figure 9, the valence band is mainly dominated by O-2p orbitals, while the conduction band is mainly formed by V-3d states.

**Figure 9:**
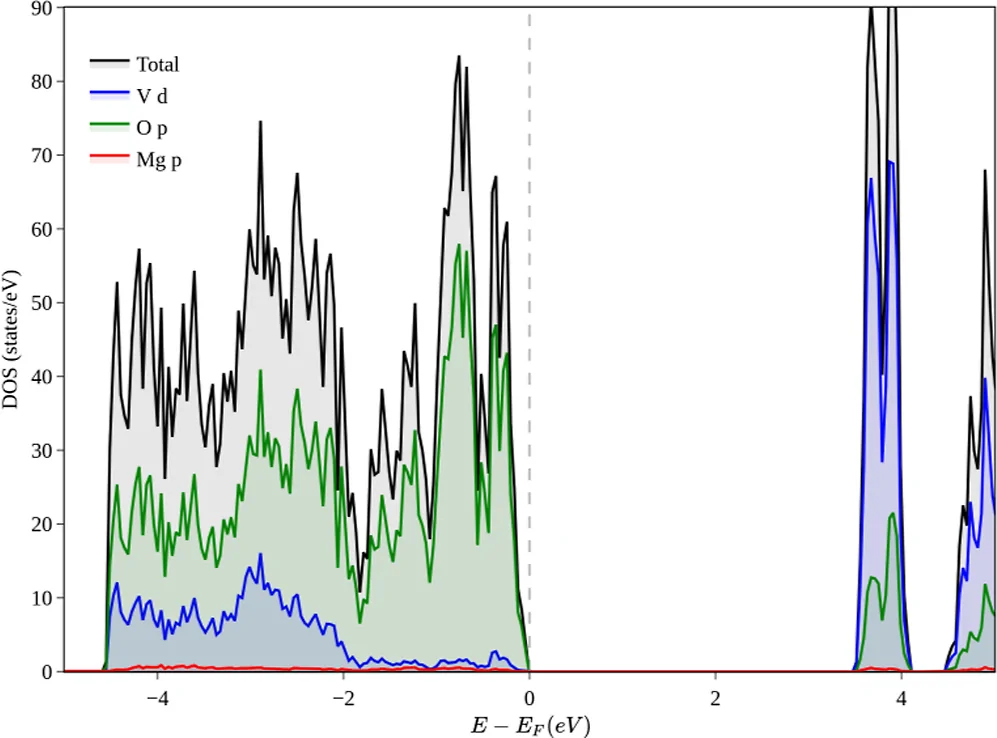
Total and projected density of states for Mg_3_V_2_O_8_.

The mechanical response of the system was then explored by evaluating its elastic properties. Although the SCAN functional performs well in predicting structural parameters and electronic band gaps, it shows deviations in describing the mechanical response, particularly anisotropic compressibility. To further assess this behavior, both the SCAN and B3LYP functionals were used to compute the full elastic tensor.

For orthorhombic crystals, there are nine independent elastic constants, denoted as *C*_*ij*_, which are listed in Table S2. To assess the mechanical stability, the calculated elastic constants were tested against the Born stability criteria^60^ for orthorhombic symmetry: *C*_11_ > 0, *C*_22_ > 0, *C*_33_ > 0, *C*_44_ > 0, *C*_55_ > 0, *C*_66_ > 0, (*C*_11_ + *C*_22_ – 2*C*_12_) > 0, (*C*_11_ + *C*_33_ – 2*C*_13_) > 0, (*C*_22_ + *C*_33_ – 2*C*_23_) > 0.

Based on the full elastic tensor, the bulk modulus (*B*), shear modulus (*G*), Young’s modulus (*E*), and Poisson’s ratio (ν) were calculated using the Voigt, Reuss, and Hill averaging schemes.^61–63^ The compiled values are presented in Table S3. The bulk modulus suggests that the material is relatively stiff with low compressibility. This is in good agreement with previous reports,^32^ further supporting the reliability of the applied computational approach. Furthermore, the calculated Debye temperature of 734 K agrees well with similar ionic systems,^64^ reinforcing the material’s mechanical rigidity.

To further investigate anisotropic mechanical behavior, we calculated the shear anisotropic factors (*A*_1_, *A*_2_, *A*_3_) for the principal crystallographic planes using the following expressions:

$$A_{1}=\frac{4C_{44}}{C_{11}+C_{33}-2C_{13}},\left(\text{for}\;the\;\left\{100\right\}\;plane\right)$$

$$A_{2}=\frac{4C_{55}}{C_{22}+C_{33}-2C_{23}},\left(\text{for}\;the\;\left\{010\right\}\;plane\right)$$

$$A_{3}=\frac{4C_{66}}{C_{11}+C_{22}-2C_{12}},\left(\text{for}\;the\;\left\{001\right\}\;plane\right)$$

Deviations of these anisotropy factors from unity reflect directional dependence in the shear response, which may influence the mechanical performance under applied stress. The values of *A*_1_, *A*_2_, and *A*_3_, as listed in Table S4, differ markedly from unity, reflecting a clear anisotropy in elastic behavior, in agreement with experimental findings. Calculated values for compressibility and shear anisotropy (*A*_comp_ and *A*_shear_) indicate that Mg_3_V_2_O_8_ has anisotropic compressibility, which agrees with experimental results of the current study.

Compressibility is inherently anisotropic in many crystalline materials, and this anisotropy is often reflected in both the mechanical stiffness and thermal expansion behavior. Notably, the two functionals predict different trends in the elastic anisotropy. B3LYP yields a lower *C*_11_ compared with *C*_33_ and *C*_22_, suggesting relatively higher compressibility along the *a*-axis. This trend is consistent with experimental thermal expansion data, where the *a*-axis shows the strongest response. In contrast, SCAN predicts a different anisotropic ordering of elastic constants, indicating that compressibility trends are more sensitive to the choice of the exchange–correlation functional. Overall, B3LYP provides better qualitative agreement with the experimentally observed anisotropic behavior under ambient conditions.^32^

The differences between SCAN and B3LYP results arise from their different treatments of the exchange–correlation effects. SCAN, as a *meta*-GGA functional, includes kinetic energy density dependence and provides an improved description of localized electronic behavior, which is beneficial for the structural and electronic properties. In contrast, B3LYP includes a fraction of exact Hartree–Fock exchange, which can improve the description of bonding-related properties such as force constants and elastic response. These methodological differences naturally lead to variations in predicted mechanical properties.^31^

To examine the intrinsic structural response of the Mg_3_V_2_O_8_ framework to Nb incorporation, 3.125, 6.25, and 9.375% substitution of Nb at the V site was investigated using an idealized single-phase 2 × 1 × 2 supercell containing 48 Mg, 128 O, and 32 V atoms. Nb atoms were placed as far apart as possible to minimize defect–defect interactions (see Figure S8). The optimized lattice parameters, relative lattice changes, unit-cell volume, and band gap values as a function of Nb concentration are summarized in Table 6.

**Table 6:** SCAN-DFT-Optimized Lattice Parameters (*a*, *b*, *c*), Unit-Cell Volume (*V*), Relative Lattice Changes $\left(\frac{\Delta a}{a_{0}},\;\frac{\Delta b}{b_{0}},\;\frac{\Delta c}{c_{0}}\right)$ , and Band Gap Values for Pure and Nb-Substituted Mg_3_V_2_O_8_ Supercells

%	*a* (Å)	*b* (Å)	*c* (Å)	*V* (Å^3^)	$\frac{\Delta a}{a_{0}}$	$\frac{\Delta b}{b_{0}}$	$\frac{\Delta c}{c_{0}}$	band gap (eV)
0	6.008	11.429	8.27	567.91	0	0	0	3.57
3.125	6.017	11.444	8.284	570.485	0.001498	0.001312	0.001693	3.3664
6.25	6.02	11.458	8.291	571.887	0.001997	0.002537	0.002539	3.3691
9.375	6.022	11.471	8.298	573.3	0.00233	0.003675	0.003386	3.372

The calculations reveal a systematic and monotonic expansion of the lattice upon Nb substitution, accompanied by an increase in unit-cell volume. This behavior reflects the larger effective ionic size of Nb^5+^ compared to V^5+^ and the structural relaxation of the Mg_3_V_2_O_8_ lattice under chemical substitution within an idealized single-phase solid solution. Importantly, the continuous increase of all lattice parameters up to 9.375% Nb suggests that Nb incorporation within the investigated concentration range does not exhibit a tendency toward saturation driven by local lattice strain. The lattice expansion is anisotropic, with relative changes following the trend $\frac{\Delta b}{b_{0}}∼\frac{\Delta c}{c_{0}}>\frac{\Delta a}{a_{0}}$ , indicating preferential structural response along the *b* and *c* crystallographic directions.

To further examine the influence of possible thermodynamic contributions on the experimentally observed solubility limit, a 0 K tie-line decomposition analysis was performed. In this analysis, the total energies of Mg_3_V_2(1–*x*)_Nb_2*x*_O_8_ were compared with phase-separated reference states. In the V-rich limit, the reference state was Mg_3_V_2_O_8_, while in the Nb-rich limit it was MgNb_2_O_6_ + 2MgO (see Figure S9). MgO was included to maintain stoichiometric balance. The relative stability of the doped configurations was quantified using the energy difference with respect to this decomposition pathway, defined as:

$$\Delta E_{decomp}\left(x\right)=E_{doped}\left(x\right)-\left[\left(1-x\right)E_{pure}+xE_{ref}\right]$$

Δ*E*_decomp_ represents the decomposition energy relative to the phase-separated reference states. *E*_doped_ is the total energy of Mg_3_V_2(1–*x*)_Nb_2*x*_O_8_, *E*_pure_ is the energy of Mg_3_V_2_O_8_, and *E*_ref_ corresponds to the energy of the Nb-rich competing phase.

The calculated decomposition energies are positive for all investigated Nb concentrations, indicating that the formation of phase-separated states is energetically favored over the ideal single-phase solid solution at 0 K. Although the decomposition energies do not increase monotonically over the entire Nb concentration range, their consistently positive values indicate an overall thermodynamic tendency toward phase separation. The calculated decomposition energies reach values of up to approximately 1.11 eV/f.u., further supporting the presence of a thermodynamic driving force for decomposition.

At the same time, the optimized DFT structures exhibit continuous monotonic lattice expansion with increasing Nb concentration and do not show an intrinsic structural saturation behavior. Therefore, comparison with experiments suggests that the observed saturation of lattice parameters near 4% Nb may be influenced by thermodynamic competition with Nb-rich secondary phases, rather than arising solely from strain effects within the host lattice.

It should also be noted that the present calculations correspond to 0 K conditions and do not explicitly include configurational entropy or finite-temperature stabilization effects. Such effects may partially stabilize dilute Nb incorporation at low concentrations during the synthesis.

## Conclusion

This study provides a detailed investigation of the structural, thermal, and elastic properties of the Mg_3_V_2(1–*x*)_Nb_2*x*_O_8_ solid solution, focusing on the effects of Nb substitution within a kagome staircase framework. Powder X-ray diffraction confirmed the formation of a orthorhombic structure (*Cmca*) for compositions up to *x* = 0.04 with trace of secondary phase, Mg_2_V_2_O_7_. Substitution of Nb^5+^ for V^5+^ leads to an increase in lattice parameters *a*, *b*, and *c* and consequently the unit cell volume, supporting the successful incorporation of Nb into the Mg_3_V_2_O_8_ lattice. The experimental results indicate a solid-solution limit of approximately *x* ∼ 0.04, beyond which further Nb addition leads to the formation of a secondary MgNb_2_O_6_ phase. Although the calculations do not directly establish a thermodynamic solubility limit, they support an increasing thermodynamic tendency toward phase separation with increasing Nb content. Experimentally, Nb substitution increases the distortion index of the VO_4_ tetrahedra, while the MgO_6_ octahedra remain unaffected. Variable-temperature XRD data (298–1273 K) confirm the structural stability of the material over a wide temperature range, with no phase transitions or symmetry changes observed. Lattice parameters exhibit smooth, but anisotropic thermal expansion, with the largest expansion along the *a*-axis. Elastic property calculations based on the full elastic tensor reveal a similar anisotropy in compressibility (κ_*a*_ > κ_*c*_ > κ_*b*_), consistent with experimental trends. Notably, the B3LYP functional effectively captures this mechanical anisotropy, demonstrating its suitability for describing the structural response under thermal or mechanical perturbations. Furthermore, the calculated high Debye temperature of 734 K suggests a strong lattice rigidity. Overall, the coupling between Nb-induced local structural distortion, anisotropic lattice response, and electronic structure suggests that this class of materials could be of interest for optoelectronic devices, thermally stable ceramics, and nonlinear optical materials. Furthermore, the formation of mixed (V,Nb)­O_4_ oxyanion units may provide a useful strategy for tuning the properties of other kagome-staircase and related orthovanadate materials.

## Supplementary Material



## Notes

Additional crystallographic and computational data, including Rietveld refinement, structural variation analyses, supercell models of pure and Nb-doped Mg_3_V_2_O_8_, 0 K tie-line decomposition analysis, and calculated elastic and thermodynamic properties of the studied materials (PDF)

## #

Deceased October 1, 2025.
