# Supplementary material for: Solubility Limit and Thermostructural Properties of Kagome Mg3V2(1–x)Nb2x O8: A Joint Diffraction and Spectroscopic Study
Source: Inorg Chem. 2026 Aug 18;65(34):19682–94. doi: 10.1021/acs.inorgchem.6c00969 (PMC13536936; doi:10.1021/acs.inorgchem.6c00969)
Supplement: Supplementary file 1 [file ic6c00969_si_001.pdf]

Supporting information

Solubility limit and thermostructural properties of kagome $Mg_3V_{2(1-x)}Nb_{2x}O_8$: A joint diffraction and spectroscopic study

Houri S. Rahimi Mosafer*[1], Asiye Shokri[2,3], Iraida N. Demchenko[3,4], Anna Wolska[1], Marcin Klepka[1], Aleksandra Drzewiecka-Antonik[1], Alexey Shekhovtsov[5], Miron Kosmyna[5], Roman Minikayev[1], and Wojciech Paszkowicz[†1]

[1]Institute of Physics, Polish Academy of Science, al. Lotnikow 32/46, 02-691 Warsaw, Poland

[2]International Research Centre Magtop, Institute of Physics, Polish Academy of Sciences, Aleja Lotnikow 32/46, PL-02668 Warsaw, Poland

[3]Institute of Plasma Physics and Laser Microfusion, ul. Hery 23, 01-497 Warsaw, Poland

[4]The Centre for Advanced Materials and Technologies, CEZAMAT at the Warsaw University of Technology, 19 Poleczki St, 02-822 Warsaw, Poland

[5]Institute for Single Crystals, NAS of Ukraine, Nauky Ave. 60, 61001Kharkov, Ukraine

*Email: rahimi@ifpan.edu.pl

† Deceased October 1, 2025.

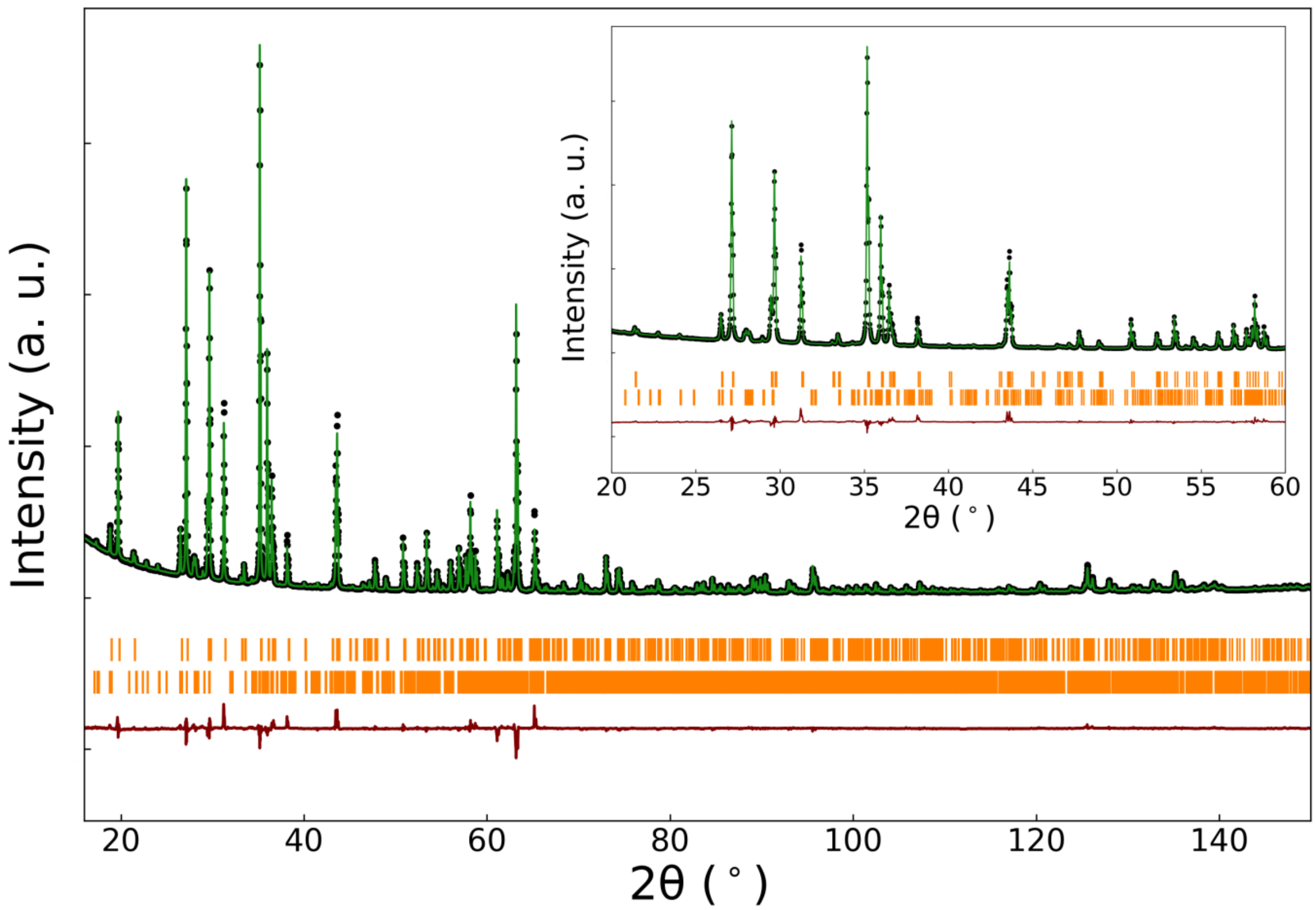

Figure S1. Illustration of Rietveld refinement of $Mg_3V_2O_8$. Black circles correspond to the experimental data, the green line to the calculated profile, orange ticks to Bragg reflections (upper: $Mg_3V_2O_8$-main phase and lower: $Mg_2V_2O_7$-minor phase) and the red line to the residual.

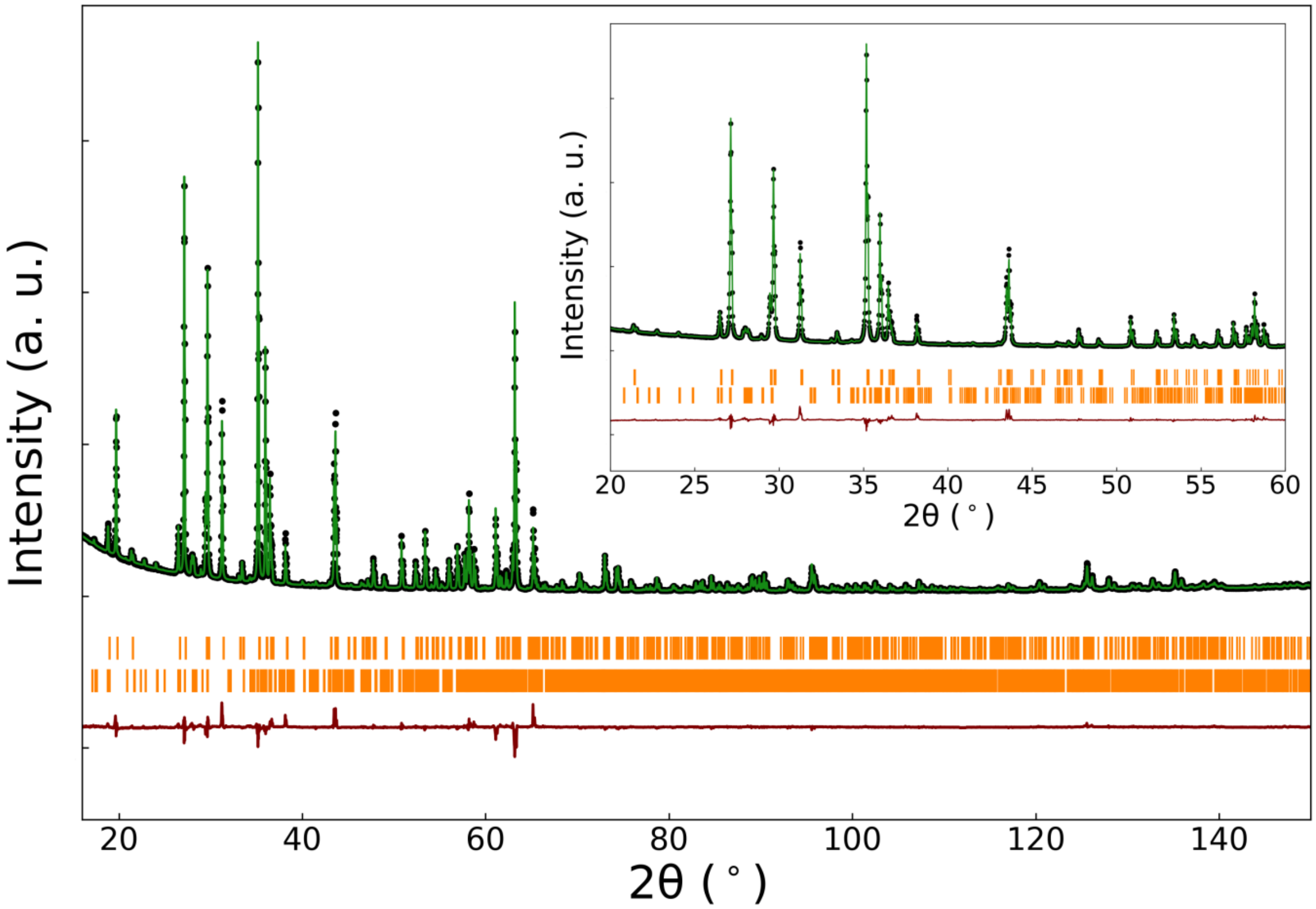

Figure S2. Illustration of Rietveld refinement of $Mg_3V_{1.98}Nb_{0.02}O_8$. Black circles correspond to the experimental data, the green line to the calculated profile, orange ticks to Bragg reflections (upper: $Mg_3V_{1.98}Nb_{0.02}O_8$-main phase and lower: $Mg_2V_2O_7$-minor phase) and the red line to the residual.

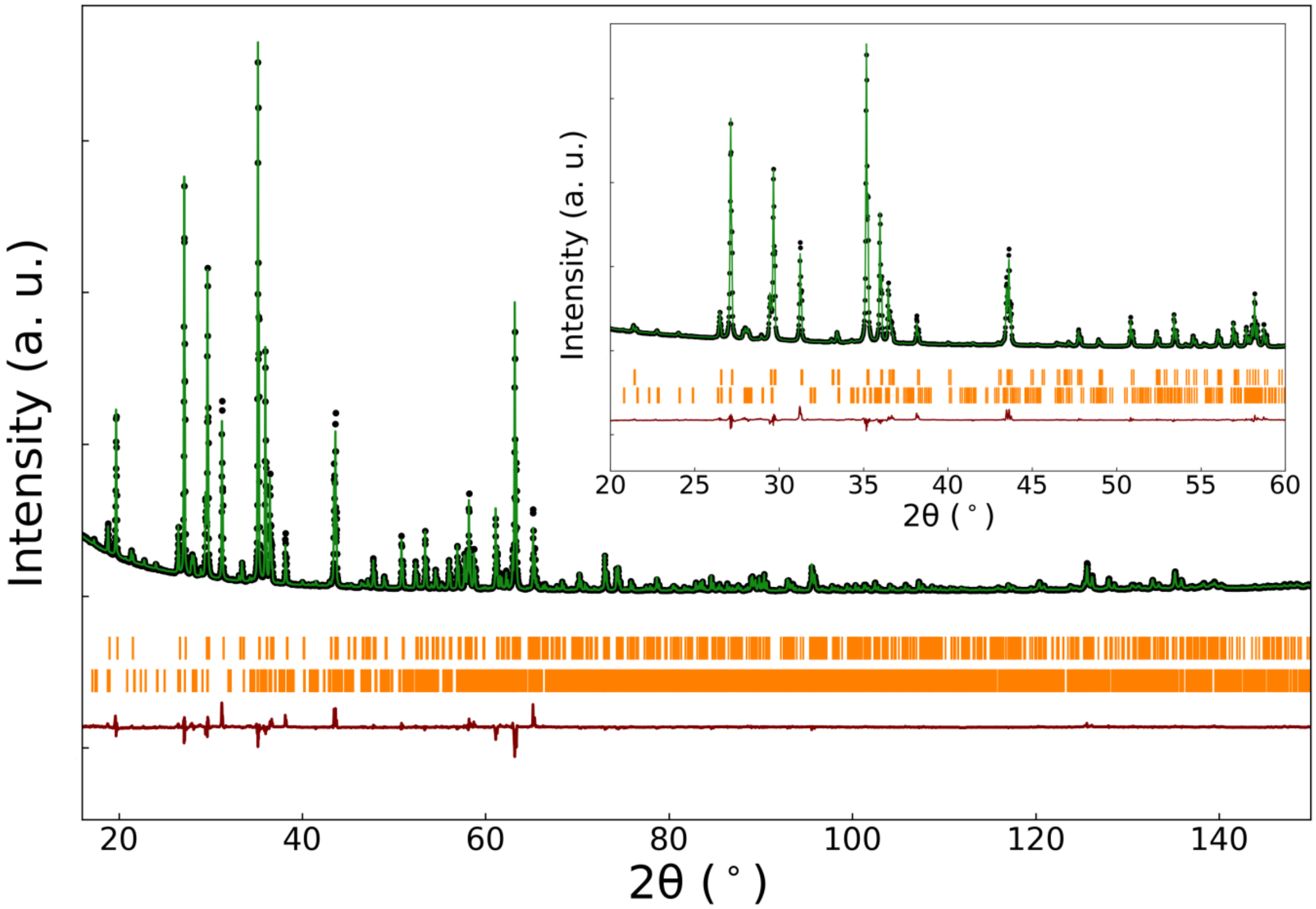

Figure S3. Illustration of Rietveld refinement of $Mg_3V_{1.94}Nb_{0.06}O_8$. Black circles correspond to the experimental data, the green line to the calculated profile, orange ticks to Bragg reflections (upper: $Mg_3V_{1.94}Nb_{0.06}O_8$-main phase and lower: $Mg_2V_2O_7$-minor phase) and the red line to the residual.

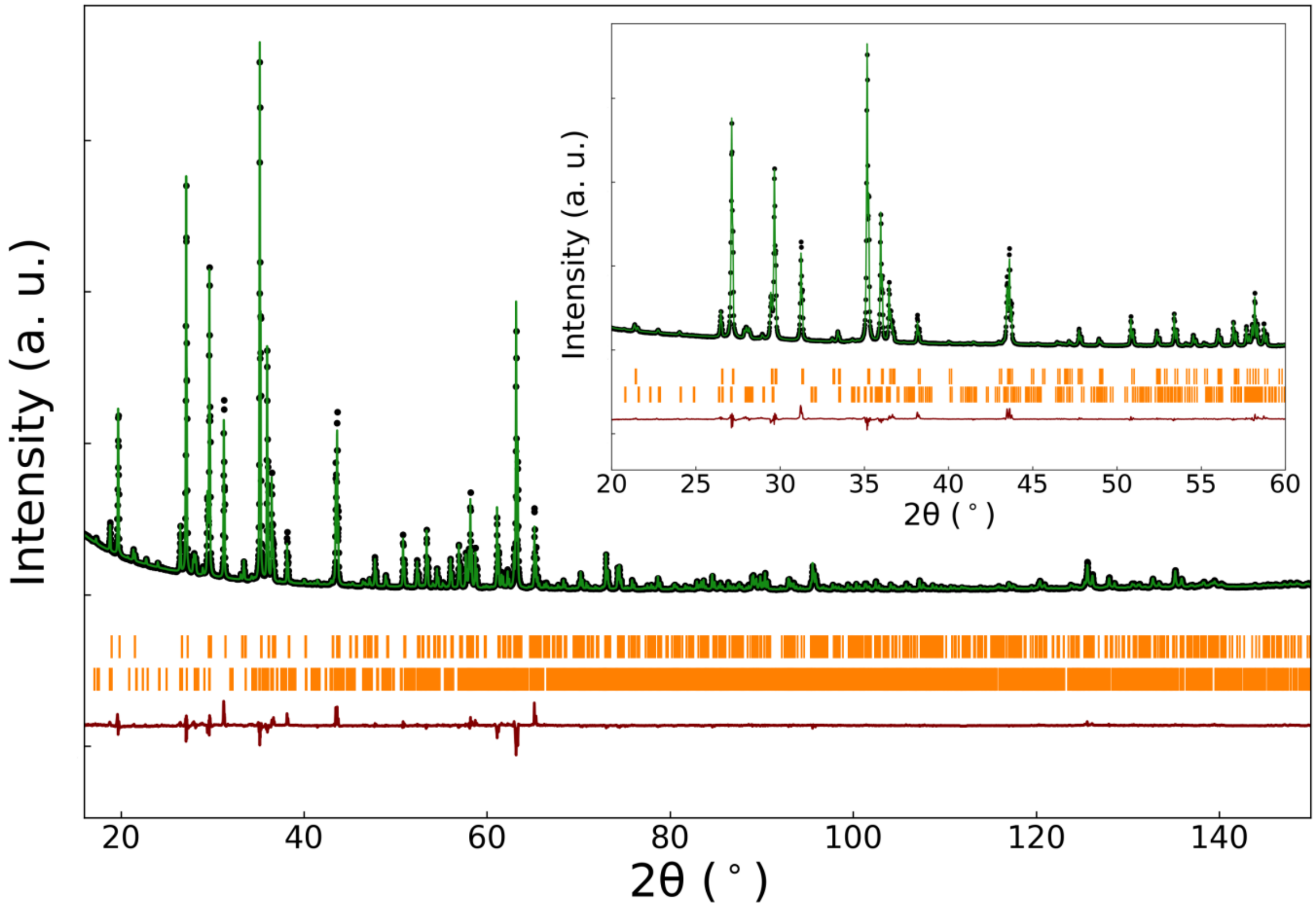

Figure S4. Illustration of Rietveld refinement of $Mg_3V_{1.92}Nb_{0.08}O_8$. Black circles correspond to the experimental data, the green line to the calculated profile, orange ticks to Bragg reflections (upper: $Mg_3V_{1.92}Nb_{0.08}O_8$-main phase and lower: $Mg_2V_2O_7$-minor phase) and the red line to the residual.

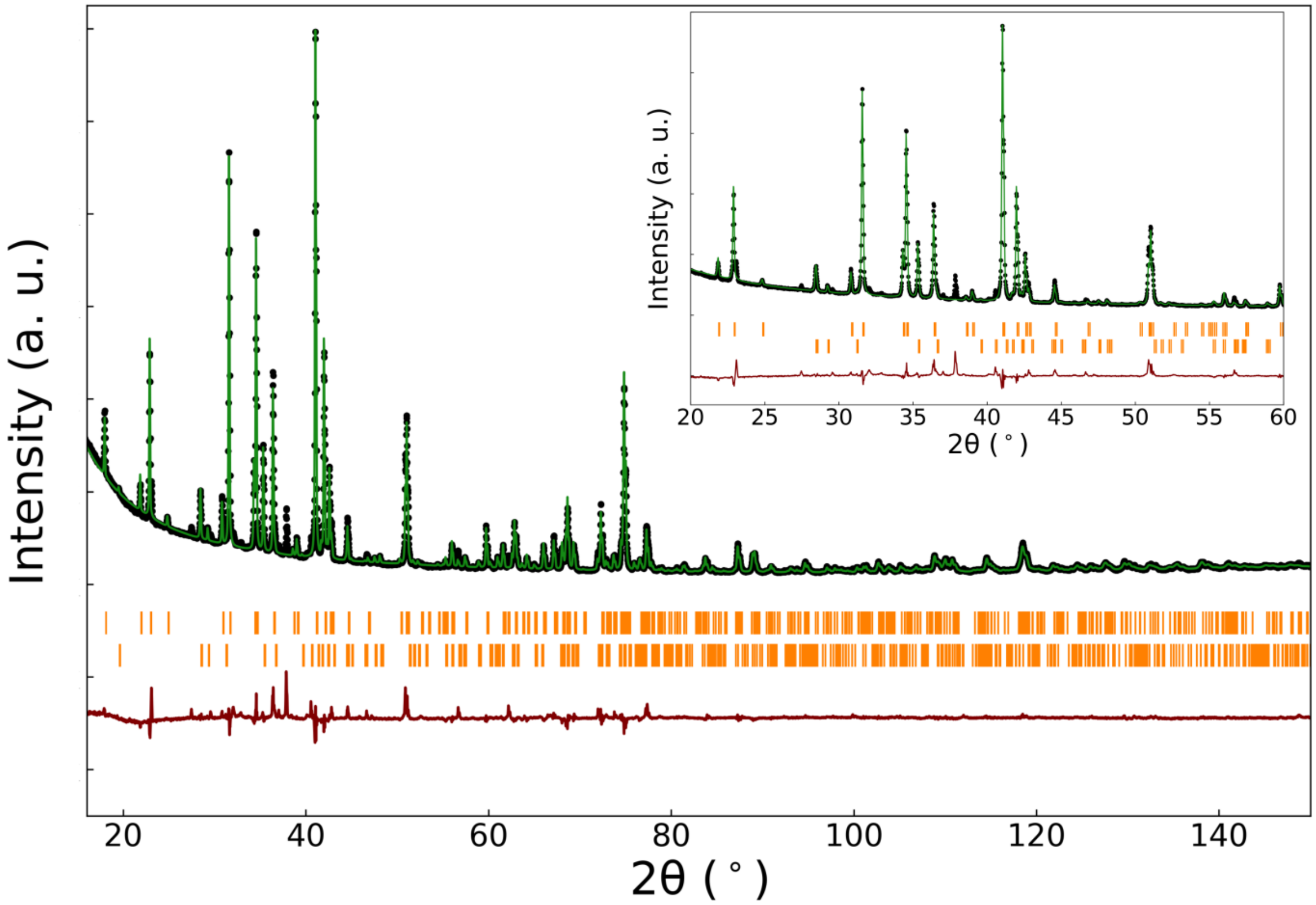

Figure S5. Illustration of Rietveld refinement of $Mg_3V_{1.80}Nb_{0.2}O_8$. Black circles correspond to the experimental data, the green line to the calculated profile, orange ticks to Bragg reflections (upper: $Mg_3V_{1.80}Nb_{0.2}O_8$-main phase and lower: $MgNb_2O_6$-minor phase) and the red line to the residual.

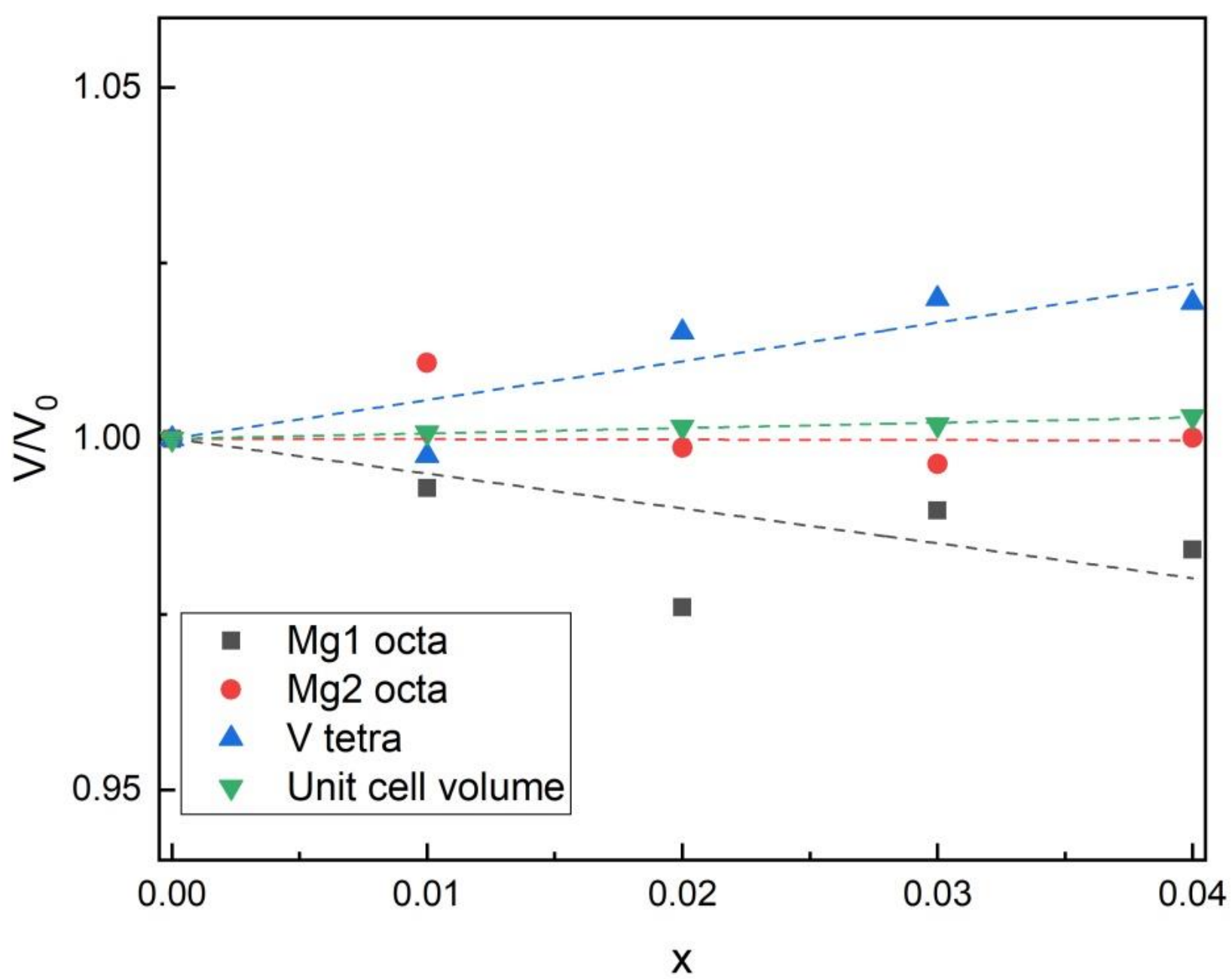

Figure S6. Variation of normalized polyhedral volumes (V/V_0) for Mg(1), Mg(2), and V sites, as well as the unit cell volume, as a function of Nb substitution level (*x*).

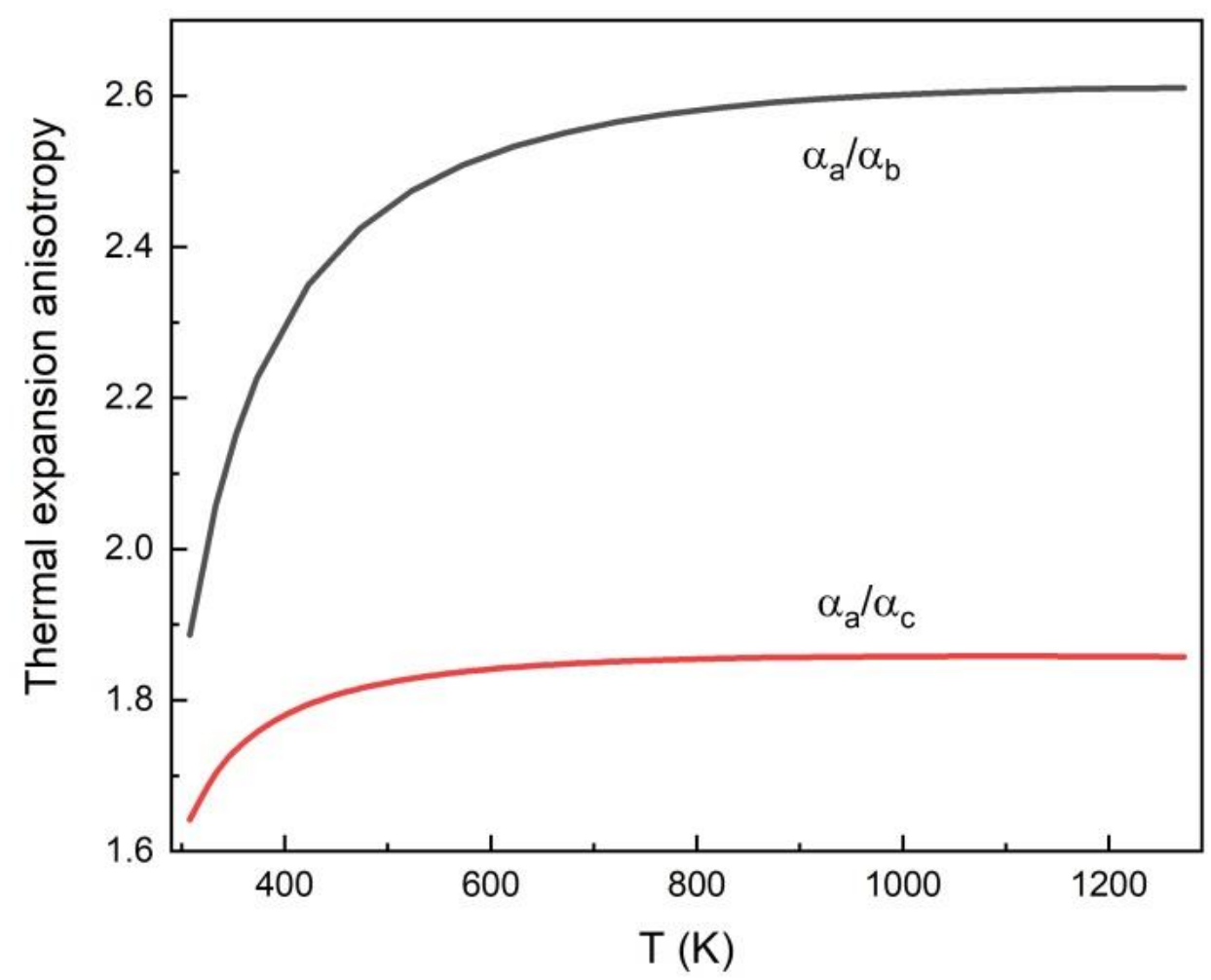

Figure S7. Variation of thermal expansion anisotropy with temperature for $Mg_3V_2O_8$.

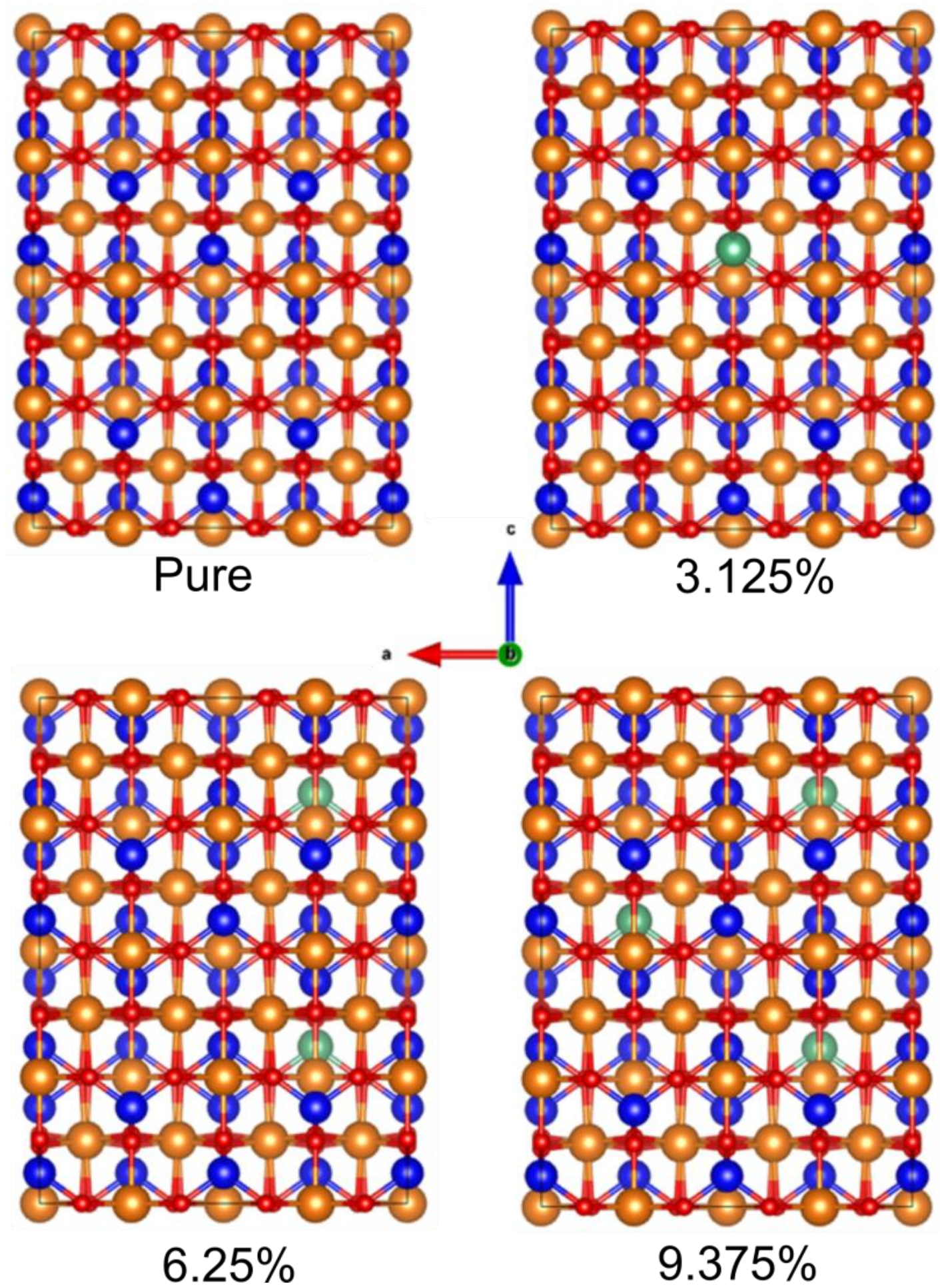

Figure S8. Supercell models of pure and Nb-doped $Mg_3V_2O_8$ used in the DFT calculations. Mg atoms are shown in orange, V in blue, Nb in light green, and O in red.

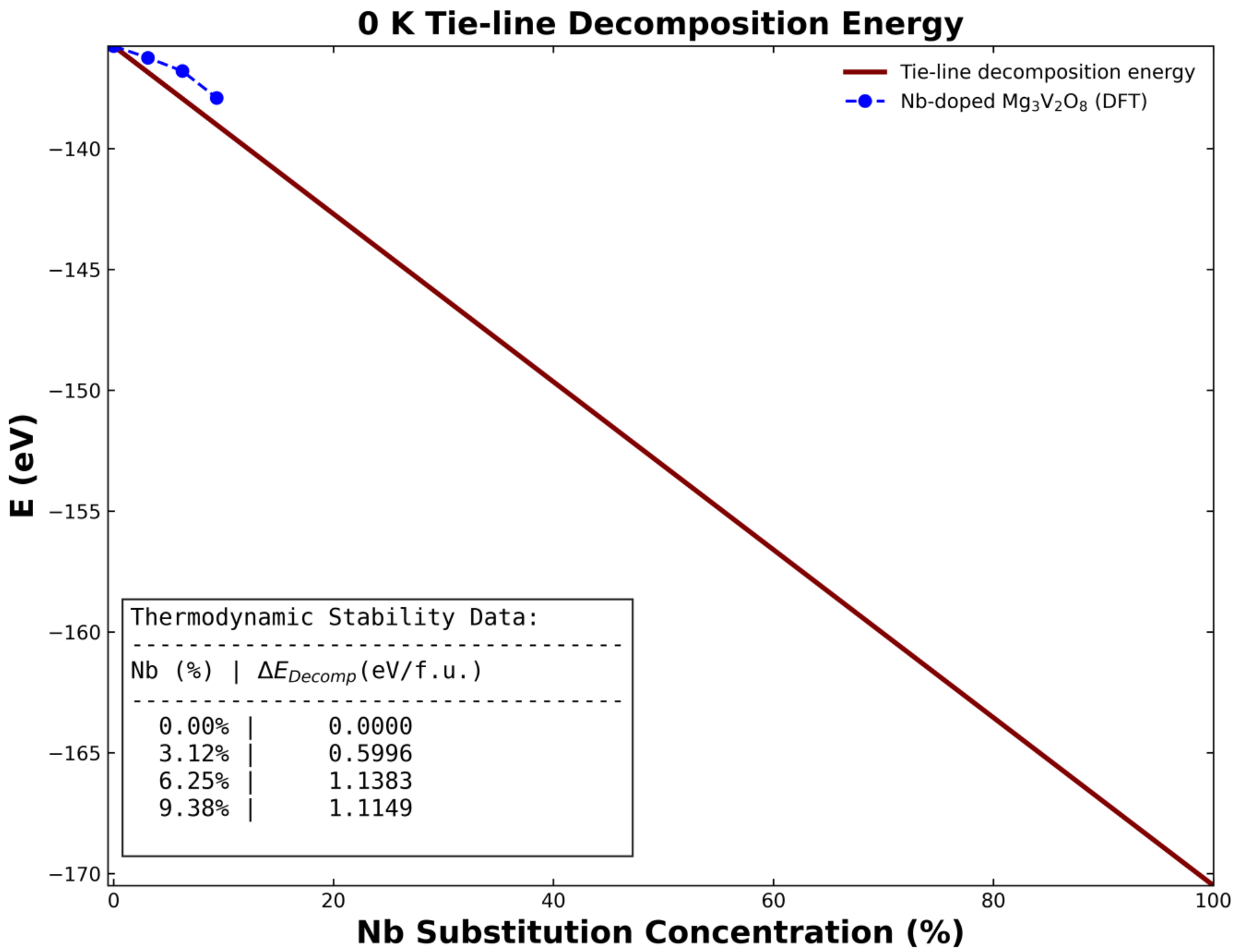

Figure S9. 0 K tie-line decomposition analysis of $Mg_3V_{2(1-x)}Nb_{2x}O_8$. The maroon dashed line represents the tie-line connecting pristine $Mg_3V_2O_8$ and the Nb-rich secondary phase reference $MgNb_2O_6$ + 2MgO. Blue circles denote the DFT-calculated decomposition energies of the idealized single-phase solid solution relative to the phase-separated reference state. The positive decomposition energies indicate an increasing thermodynamic tendency toward phase separation with increasing Nb concentration.

Table S1. Phase composition (wt.% from Rietveld refinement) of $Mg_3V_{2(1-x)}Nb_{2x}O_8$ samples with Nb content (*x*), together with the Rietveld refinement reliability factors (Rp and Rwp).

x	Phase fraction (wt.%)			R_p (%)	R_{wp} (%)
	$Mg_3V_2O_8$	$Mg_2V_2O_7$	$MgNb_2O_6$		
0	95.5(3)	4.50(6)	-	3.34	4.73
0.01	93.9(4)	6.10(8)	-	3.70	5.11
0.02	95.8(3)	4.20(5)	-	2.98	3.96
0.03	95.6(4)	4.40(7)	-	3.76	5.34
0.04	96.8(3)	3.20(6)	-	3.10	4.29
0.1*	95.4(6)	-	4.61(5)	4.06	6.11

*x = 0.1 refer to the nominal composition, while the true Nb content corresponds to the solubility limit.

Table S2. Calculated elastic constants (GPa) for $Mg_3V_2O_8$

Method	C_{11}	C_{12}	C_{13}	C_{22}	C_{23}	C_{33}	C_{44}	C_{55}	C_{66}
SCAN	265.01	68.997	91.817	258.37	84.636	257.38	75.94	103.08	76.49
B3LYP	234.483	75.842	83.585	268.645	92.959	243.966	73.452	94.815	73.824

Table S3. Calculated isotropic bulk modulus B(GPa), shear modulus G (GPa), Young's modulus E (GPa) and Poisson's ratio for $Mg_3V_2O_8$

Method	B_R (GPa)	B_V (GPa)	B_H (GPa)	G_R (GPa)	G_V (GPa)	G_H (GPa)	E (GPa)	ν	B/G
SCAN	140.99	141.30	141.14	85.26	86.79	86.03	214.50	0.247	1.64
B3LYP	138.451	139.10	138.774	80.451	81.40	80.925	204.34	0.26	1.715

Table S4. The calculated sound velocities (v_t, vl, v_m), the Debye temperatures anisotropic factors for $Mg_3V_2O_8$

Material	A_1	A_2	A_3	A_{comp}	G_{shear}	v_t	v_l	v_m	θ_D (K)	Ref.
$Mg_3V_2O_8$ (SCAN)	0.896	1.19	0.794	0.079	0.277	4928.73	8499.78	5469.73	734	This work
$Mg_3V_2O_8$ (B3LYP)	0.944	1.161	0.840	0.075	0.282	4704.88	8214.26	5226.92	708.9	This work
$Ni_3V_2O_8$	0.949	0.918	1.044	0.283	0.161	3523	6622	3937	530	[1]
$Ni_3V_2O_8$	—	—	—	—	—	—	—	—	600	[2]
$Mn_3V_2O_8$	0.841	1.090	1.151	1.811	2.620	3246	5974	3622	472	[1]
$Co_3V_2O_8$	—	—	—	—	—	—	—	—	550	[2]

References:

[1] H. Koc, S. Palaz, A. M. Mamedov, and E. Ozbay, "Electronic and elastic properties of the multiferroic crystals with the Kagome type lattices -Mn3V2O8 and Ni3V2O8: First principle calculations," *Ferroelectrics*, vol. 544, no. 1, pp. 11–19, 2019, doi: 10.1080/00150193.2019.1598178.

[2] R. C. Rai *et al.*, "High-energy magnetodielectric effect in kagome staircase materials," *Phys. Rev. B - Condens. Matter Mater. Phys.*, vol. 76, no. 17, pp. 1–12, 2007, doi: 10.1103/PhysRevB.76.174414.
